# Cell Cycle Commitment and the Origins of Cell Cycle Variability

**DOI:** 10.3389/fcell.2021.698066

**Published:** 2021-07-23

**Authors:** Robert F. Brooks

**Affiliations:** ^1^Molecular and Clinical Sciences Research Institute, St George’s, University of London, London, United Kingdom; ^2^Department of Anatomy, King’s College London, London, United Kingdom

**Keywords:** cell cycle variability, restriction point, bistable switches, RB-E2F switch, APC/C^CDH1^ switch, transition probability, quiescence, heterogeneity

## Abstract

Exit of cells from quiescence following mitogenic stimulation is highly asynchronous, and there is a great deal of heterogeneity in the response. Even in a single, clonal population, some cells re-enter the cell cycle after a sub-optimal mitogenic signal while other, seemingly identical cells, do not, though they remain capable of responding to a higher level of stimulus. This review will consider the origins of this variability and heterogeneity, both in cells re-entering the cycle from quiescence and in the context of commitment decisions in continuously cycling populations. Particular attention will be paid to the role of two interacting molecular networks, namely the RB-E2F and APC/C^CDH1^ “switches.” These networks have the property of bistability and it seems likely that they are responsible for dynamic behavior previously described kinetically by Transition Probability models of the cell cycle. The relationship between these switches and the so-called Restriction Point of the cell cycle will also be considered.

## Introduction

When starved of growth factors, normal mammalian cells cease proliferating and arrest in a quiescent state outside the cell cycle, now commonly referred to as G0 (Holley and Kiernan, 1968; Burk, 1970). On re-addition of growth factors (typically in the form of serum), the cells resume cycling but only after a long lag comparable to the duration of the entire cell cycle of rapidly proliferating cells (Burk, 1970; Temin, 1971). This lag is independent of the concentration of growth factors or serum, even though these have widespread effects on cellular growth (mass increase) and metabolism (Temin, 1971; Brooks, 1975, 1976). Following the lag, the cells start entering S phase asynchronously, at a rate determined by the level of growth factors (Brooks, 1975, 1976). If the growth factors are removed again at any point, even before the end of the lag, many cells continue on into S phase and mitosis in the absence of further stimulation (Todaro et al., 1965; Burk, 1970; Temin, 1971; Brooks, 1976). Cells therefore appear to become committed to re-enter the cell cycle sometime before they reach S phase. This point of commitment, after which subsequent progress through the cell cycle becomes independent of growth factors, is known as the Restriction Point (Pardee, 1974). For normal, growth factor-dependent cells, the Restriction Point is widely regarded as a critical decision point that must be passed in each and every cell cycle (Planas-Silva and Weinberg, 1997). Underscoring its importance, regulation of this transition appears to be defective in most if not all cancers (Malumbres and Barbacid, 2009).

In recent years there has been a great deal of progress in understanding the molecular details of the Restriction Point – see Pennycook and Barr (2020) for an excellent recent review. However, what determines the timing of the Restriction Point remains far from clear. When stimulated from quiescence, some cells (even in clonal populations) require much higher levels of growth factors than others to be triggered into S phase (Brooks et al., 1984). Even with maximal stimulation, the cells enter S phase at different times over many hours, indicating asynchronous passage of the Restriction point (Brooks, 1975, 1976). This asynchrony and heterogeneity is often regarded merely as a nuisance, limiting the utility of serum starvation/refeeding as a means to synchronize the cell cycle. However, an alternative view is that the variability may actually be saying something about the way in which cell cycle commitment is regulated. At the very least, understanding the origin of the variability is essential to any complete understanding of cell cycle regulation. In this article, some of the causes of this variability will be explored, with a particular focus on the RB-E2F and APC/C^CDH1^ bistable switches (Stallaert et al., 2019; Pennycook and Barr, 2020). These have the property of excitability and increasingly seem likely to lie behind key all-or-none commitment steps in the cell cycle.

## The RB-E2F Bistable Switch

**FIGURE 1:**
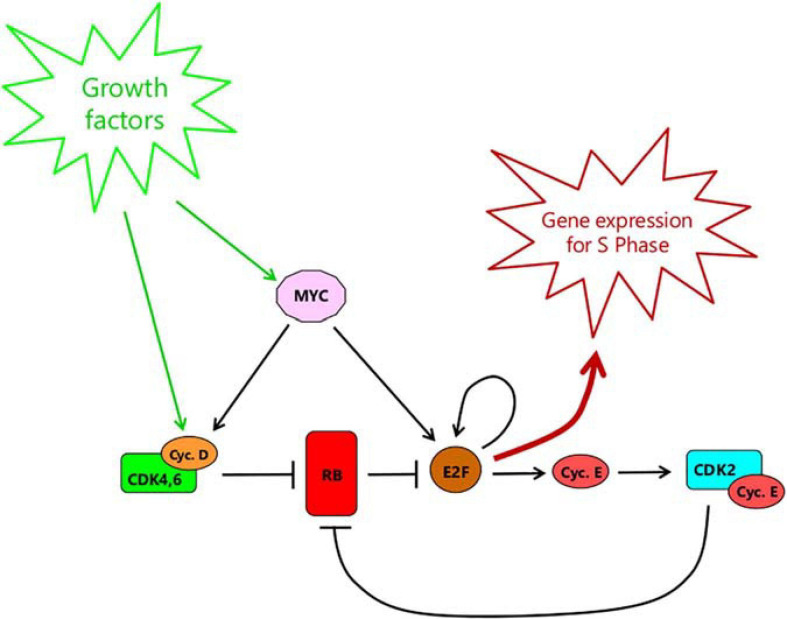
The RB-E2F bistable switch, as outlined by Yao et al. (2008). RB represents the pocket protein family consisting of RB itself, p130 and p107. E2F refers to all activator forms, namely E2F1, E2F2, and E2F3a complexed with a dimerization partner DP1 or DP2. This simplified view of the RB-E2F pathway continues to be useful and conceptually valid. However, see Box 1 for discussion of the distinctive roles of the different RB family members that underlie the pathway.

It is now widely accepted (Planas-Silva and Weinberg, 1997; Johnson and Skotheim, 2013; Stallaert et al., 2019; Pennycook and Barr, 2020) that passage of the Restriction Point is regulated by the RB-E2F pathway (Figure 1). RB in this context refers to a family of so-called pocket proteins that includes RB itself, the product of the retinoblastoma susceptibility gene, together with p130 and p107 (Cobrinik, 2005). RB family proteins bind to members of the E2F family of transcription factors, repressing the expression of E2F-target genes either directly or through recruitment of chromatin modifiers such as histone deacetylase (Cobrinik, 2005; Choi and Anders, 2014). Of particular importance are E2F1-3a, needed for the expression of many genes required for DNA synthesis and cell cycle progression (Bertoli et al., 2013). Indeed, knock-out of these E2Fs prevents cell cycle re-entry from quiescence (Wu et al., 2001) while ectopic overexpression alone is sufficient to drive quiescent cells into S phase (Johnson et al., 1993). Likewise, elimination of E2F repression by knock-out of all three RB family members prevents cell cycle exit into quiescence (Sage et al., 2000). For more detail of the distinctive roles of the different Rb family proteins, see Box 1.

**BOX 1. Distinct roles of Rb family members in cell cycle-regulated gene expression.**

Rb family members are often treated as though they were largely equivalent in function (as in Figure 1), insofar as they all bind to E2F transcription factors, and this interaction is disrupted by CDK-mediated phosphorylation. This, however, is an oversimplification. Rb binds preferentially to the activator E2Fs (E2F1, E2F2, and E2F3a), whereas p107 (RBL-1) and p130 (RBL-2) associate primarily with E2F4 and E2F5, which function mainly as repressors of transcription (Cobrinik, 2005).

In quiescent cells, the level of activator E2Fs is low and the expression of genes needed for entry into the cell cycle is actively and specifically repressed by E2F4, in association with p130 (Takahashi et al., 2000; Sadasivam and DeCaprio, 2013; Schade et al., 2019). However, E2F4 and p130 do not act alone but function along with MuvB as part of the so-called DREAM complex, made up of DP, RB-like, E2F4 (or E2F5) and MuvB (Sadasivam and DeCaprio, 2013; Schade et al., 2019). The multi-subunit MuvB component binds to the CHR (cell cycle genes homology region) elements found in “late” cell cycle genes. This enables the DREAM complex to suppress both late cell cycle genes as well as E2F-dependent “early” genes, in quiescence.

After mitogenic stimulation, phosphorylation of p130 by CDK4,6/cyclin D, midway through the pre-replicative lag, leads to disruption of the DREAM complex, the dissociation of E2F4 (Schade et al., 2019) and its replacement by E2F1, E2F2, and E2F3 (Takahashi et al., 2000). This in turn allows expression of “early” cell cycle genes. Sometime after this, MuvB (presumably still bound to the CHR elements of late cell cycle genes, maintaining suppression) is joined by BMYB (itself a product of early gene expression) (Sadasivam and DeCaprio, 2013). The BMYB-MuvB complex in turn recruits FOXM1 which, after phosphorylation (probably by CDK4,6/cyclin D – Anders et al., 2011) induces expression of late cell cycle genes in G2 (Sadasivam and DeCaprio, 2013).

Although p130 and the DREAM complex contribute to the suppression of cell cycle gene expression in quiescence, RB itself (which is not able to form complexes with MuvB) appears to play a greater role (Schade et al., 2019). Quiescent cells lacking RB show a significant de-repression of cell cycle gene expression whereas cells lacking p130 do not (Schade et al., 2019). However, cells lacking both p130 and RB show a greater de-repression of cell cycle genes than cells lacking RB only, confirming that DREAM does play a part in suppression (Schade et al., 2019). The greater role of RB was attributed to the inhibition of the activator E2Fs (1-3) still needed for gene expression after repression by E2F4 is relieved. Nevertheless, Takahashi et al. (2000) were unable to detect an association of RB with the cell cycle gene promoters examined at any time after mitogenic stimulation, even after the point half-way through the pre-replicative lag when E2F4 is replaced by E2F1, E2F2 and E2F3. This is consistent more with RB sequestering the activator E2Fs away from the promoters until after its hyperphosphorylation, rather than direct promoter repression (Cobrinik, 2005). Importantly, DREAM and RB appear to regulate the same set of genes, with little evidence for differential expression (Schade et al., 2019).

The exact role of p107 continues to be unclear. Its level is highest in proliferating cells and it is the product of an early E2F-regulated gene switched on during entry into the cycle from quiescence (Schade et al., 2019). It is also upregulated in cells deficient in p130 (Schade et al., 2019). Nevertheless, although p107 is able to complex with MuvB, there was little evidence for it doing so in p130-deficient cells (Schade et al., 2019). The limited de-repression of cell cycle-regulated cells in p130-deficient cells does not therefore seem to be due to compensatory replacement of p130 by p107 in forming the DREAM complex. It is also noteworthy that siRNA knock-down of p107 in cells lacking both RB and p130 did not lead to any consistent, additional changes in gene expression (Schade et al., 2019). There is no evidence therefore for a set of genes repressed specifically by p107. Evidently, the precise contribution of p107 to cell cycle regulation remains to be determined.

Mitogenic stimulation leads to the expression of cyclin D (Figure 1), a family of three closely related proteins, D1–D3 (in mammals) whose pattern of expression is partly cell type specific and partly dependent on the signaling pathway (Sherr, 1995; Choi and Anders, 2014). Cyclin D1 is induced by the RAS-MAPK pathway in particular, but also by Wnt/β-catenin, Notch, JAK-STAT or Hedgehog signaling (Klein and Assoian, 2008; Choi and Anders, 2014). Cyclin D2 is induced by Myc (Bouchard et al., 1999), which in turn is elevated by growth factor stimulation. Cyclin D3 is less-well studied but is widely expressed and may be important in lymphoid cells (Sicinska et al., 2003). All three bind to and activate cyclin dependent kinases 4 and 6 (CDK4,6) and, seemingly being of equivalent activity, will be referred to collectively hereafter as Cyclin D, for simplicity. The active CDK4,6/Cyclin D then phosphorylates RB family proteins – its major substrates (Sherr, 1995; Choi and Anders, 2014). The precise details of phosphorylation are complex, and will be revisited later. For now, phosphorylation on multiple sites (“hyperphosphorylation”) leads to dissociation of RB proteins from E2F, allowing the latter to activate expression of target genes (Mittnacht, 1998; Bracken et al., 2004; Choi and Anders, 2014). Among these, cyclin E is of particular importance (Figure 1). Following induction, cyclin E binds to CDK2. The active CDK2-cyclin E complex then, in turn, contributes to the hyperphosphorylation of RB, further promoting the release of E2F, and further expression of cyclin E in a positive feedback loop (Yao et al., 2008; Johnson and Skotheim, 2013; Weinberg, 2013; Pennycook and Barr, 2020). The targets of E2F also include E2F1-3, contributing another positive feedback loop promoting E2F activity (Bracken et al., 2004). In addition, elevation of Myc activity in response to growth factor stimulation, besides promoting expression of Cyclin D (Bouchard et al., 1999), also directly induces expression of E2F1-3 (Leone et al., 2001) as well as promoting E2F-mediated transcription (Leung et al., 2008), further contributing to Cyclin E expression and RB suppression (Figure 1).

The positive feedback loops within the RB-E2F pathway confer the property of bistability, such that E2F activity can only be sustained at steady-state at one of two levels: either low (E2F-Off) or high (E2F-On) (Yao et al., 2008). Once above a critical threshold, E2F levels will drive inexorably upwards to the high steady state due to the positive feedback. From then on, the level of Cyclin E-CDK2 becomes sufficient to maintain the hyperphosphorylation of RB without the need for further input from Cyclin D-CDK4,6. This switch is postulated to represent passage of the Restriction Point since, after it, growth factor stimulation, and Cyclin D expression, are no longer needed to maintain E2F activity (Yao et al., 2008).

Experimental evidence that the RB-E2F pathway does indeed behave as a bistable switch came from using a green fluorescent protein (GFP)-construct under the control of the E2F1 promoter as an E2F reporter (Yao et al., 2008). After stimulating quiescent cells with a high level of serum growth factors, E2F activity rose to a high level in all cells in the population, with most going on to enter S phase. However, when stimulated with suboptimal levels of serum, the level of E2F activity became bimodal within the population, with some cells maintaining the low level of the quiescent controls, while other cells in the same population switched to a high level of E2F. This bifurcation of E2F activity within the population showed that the RB-E2F switch was able to convert continuous, graded levels of growth stimulation into all-or-none responses at the cellular level. Importantly, the cells that subsequently went on into S phase were from those that switched to high-E2F.

That some cells switch to high E2F under suboptimal conditions while others do not was attributed to cellular noise in pathway dynamics around the threshold, due either to small numbers of interacting molecules or to small differences in parameter values resulting from the previous history of the cell (size variation, for example, or local differences in cell density) (Yao et al., 2008). Later, a stochastic version of the model was indeed able to generate asynchronous switching within a population (Lee et al., 2010). Moreover, it was able to reproduce the apparently first order kinetics of entry into S phase observed experimentally, in which a constant fraction of the cells enters S phase per unit time (Brooks, 1975, 1976). These kinetics had previously been taken as evidence for the Transition Probability model of Smith and Martin (Smith and Martin, 1973) which proposed the existence of a rate-limiting commitment step in the cell cycle occurring stochastically with constant probability over time under steady-state conditions.

## The RB-E2F Switch and Heterogeneity in Exit From Quiescence

Although the stochastic version of the RB-E2F switch is able to reproduce some of the observed asynchrony in entry into S phase after stimulation from quiescence, it fails to account for other aspects of the kinetics. In particular, the model predicts that the lag between stimulation and cell cycle entry should depend on the level of stimulation (Lee et al., 2010). As already indicated, this is not the case. Rather, the lag before the first cells reach S phase is independent of growth factor (serum) concentration, even though the subsequent rate of entry into S phase varies (Brooks, 1975, 1976). Moreover, when the cells are first stimulated with a low (suboptimal) level of serum and the level raised again at the end of the first lag, there is another lag, identical to the first, before the rate of entry into S increases for a second time (Brooks, 1976). It is as though only a fraction of the population responded to the first stimulus, the rest remaining in the quiescent state until a second (higher) stimulus was able to move them out of arrest and on toward S phase. This would imply heterogeneity, even in a clonal population, such that some cells can respond to a low level of growth factors whereas others cannot.

That such heterogeneity does indeed exist was shown by later experiments in which the response to low levels of serum was followed over a much longer period. In the original experiments with observations limited to around 24 h, the rate of entry into S phase after stimulation did indeed appear first-order, following the lag, with a rate constant dependent on the level of serum. The expectation was that all cells would eventually reach S phase, if followed for long enough, even with very low levels of stimulation. This, however, is not what happens. When cells stimulated with low serum were followed over days, the rate of entry into S phase slowed, with the fraction of cells entering S phase reaching a plateau (Brooks et al., 1984). This was not because the low level of serum added was “used up” since the medium was renewed daily. Instead, it is clear that only some cells were able to respond to the low level of serum, with those responding doing so asynchronously, over many hours. However if, after reaching the plateau, the serum concentration was raised further, the previously unresponsive cells then entered S phase (Brooks et al., 1984). Thus, the non-responsive cells had not become incapable of responding, they merely needed a higher level of stimulation.

Similar results were obtained when cells were followed by timelapse microscopy rather than *^3^H*-thymidine autoradiography. However, in this case it was possible to see that some of the cells triggered to divide in response to low serum went on to divide again, in some cases several times, while many cells in the same field failed to respond at all (Brooks et al., 1984; Brooks and Riddle, 1988a). This might suggest an inherited element determining sensitivity to growth factors. However, attempts to enrich for responding cells by prolonged culture in low serum (3 weeks) were not successful (Brooks et al., 1984). Following such selection, the cells were no more responsive than the controls.

Such heterogeneity *within* a population is not predicted by the simple, stochastic version of the RB-E2F bistable switch (Lee et al., 2010). Although the inclusion of stochastic noise enables it to reproduce asynchrony in exit from quiescence, the model predicts that all cells should eventually do so, even with low levels of stimulation, given long enough, which (as already discussed) is not what happens. However, a later, extended version of the model, explicitly including a role for CDK-inhibitors such as p21 and p27, may provide an explanation (Kwon et al., 2017). Systematic varying of the model parameters showed that certain ones, in particular those affecting Cyclin-CDK activity or RB-phosphorylation status, had a marked effect on the threshold for switching (Kwon et al., 2017). Raising the threshold makes it more difficult to exit quiescence, increasing both the time required and the level of stimulation needed. Such an increase in threshold was shown to provide a compelling explanation for the well-established observation that cells do indeed go deeper and deeper into quiescence the longer they are starved of mitogenic stimulation, requiring both longer to re-enter the cycle on re-stimulation and a higher level of stimulation (Kwon et al., 2017). Later, it was shown that raising the threshold even further was able to account for a shift from deep quiescence to senescence and irreversible cell cycle arrest (Fujimaki et al., 2019; Fujimaki and Yao, 2020).

Although variation in the activation threshold of the RB-E2F switch is able to account for different levels of quiescence, in the published simulations the parameters are assumed (for simplicity) to have the same values in all cells of the population at the same time (other than stochastic noise). In practice this is unlikely to be the case. It is more probable that some parameters may vary within the population, even between adjacent cells, giving them different activation thresholds for responding to mitogenic stimulation. Of the possible parameters that might be implicated, the levels of CDK-inhibitors such as p27 and p21 are particularly attractive candidates. Simulations showed that these were among the strongest coarse tuners of the threshold in the model. In keeping with this, experimentally increased levels of p21 did indeed raise the activation threshold (Kwon et al., 2017; Heldt et al., 2018). Moreover, levels of p27 are known to increase in quiescence (Coats et al., 1996), and are heterogeneous within a population, with those cells reaching S phase after a short serum pulse being the ones with the lowest levels (Hitomi et al., 2006). It therefore seems probable that differences between individual cells in the levels of p27 or p21 contribute significantly to the heterogeneity in growth factor dependence within a quiescent population. This, of course, does not preclude the possibility that other components of the RB-E2F switch also contribute to the heterogeneity, such as levels of RB family proteins.

## Exit Into Quiescence in Cycling Populations

**FIGURE 2:**
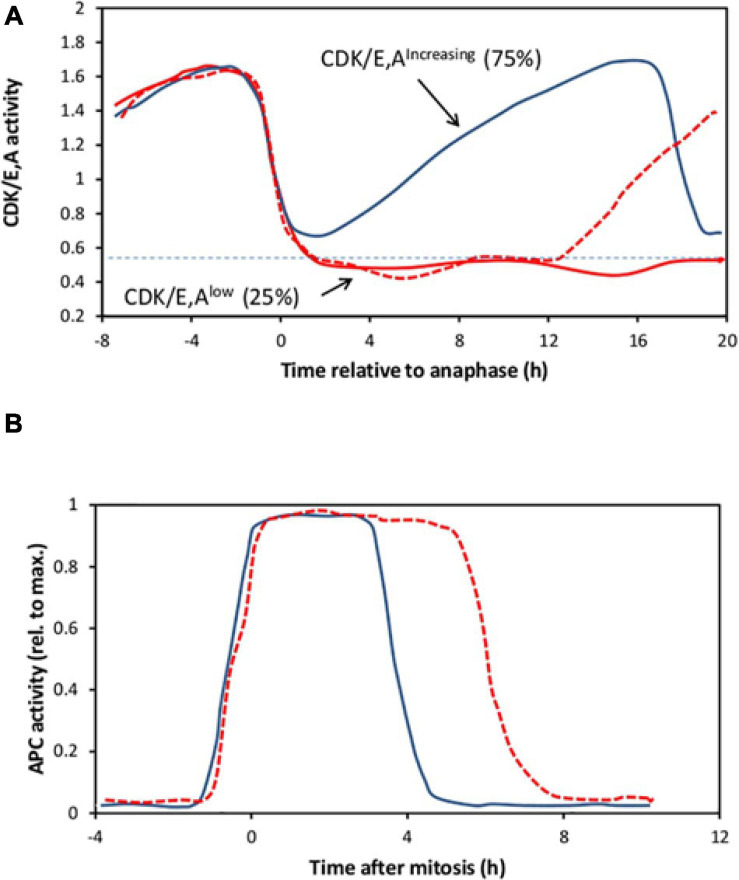
A representation of live cell imaging data for CDK or APC activity. **(A)** Cartoon illustrating three single cell traces, aligned to anaphase, to indicate the range of CDK/E,A activity changes shown by individual MCF10A cells in a continuously cycling population, based on Figure 3B of Spencer et al. (2013). Following the drop in activity after mitosis, in roughly 75% of the cells (continuous blue line), activity immediately starts rising again to a peak at the next mitosis (“CDK/E,A^*increasing*^ cells”). Although not indicated here, individual traces within this cohort, while following the same general pattern, nevertheless show a good deal of asynchrony. In the other 25% of the cells, activity falls to a low level (below the horizontal dotted line, at 2 h after anaphase, in this example). In these “CDK/E,A^*low*^ cells,” activity either remains low until the end of the experiment (continuous red line), or spontaneously starts rising again (dotted red line) as the cell switches to the CDK/E,A^*increasing*^ state. **(B)** Cartoon of two single-cell traces to illustrate APC activity in cycling MCF10A cells, aligned to mitosis, based on Figure 4I of Cappell et al. (2016). APC activity rises sharply as cells enter mitosis then drops precipitously to a very low level several hours later. The abruptness of the fall is similar in all cells, but its timing varies, indicating considerable asynchrony between individual cells.

Heterogeneity is seen not only in populations of quiescent cells responding to mitogenic stimulation. It is also a feature of continuously cycling cells. Recent developments in live cell imaging have enabled cell cycle transitions to be followed with unprecedented precision in real time. Of particular value has been the use of a fluorescent sensor based on a fragment of human DNA helicase B (DHB) that moves from the nucleus to the cytoplasm on phosphorylation (Spencer et al., 2013). This was originally thought to be selective for Cyclin-dependent kinase 2 (CDK2) but later shown to be responsive to both CDK1 and CDK2, complexed with either cyclins E or A (Schwarz et al., 2018), and is henceforth referred to here as CDK/E,A activity. Using this sensor, CDK/E,A activity is seen to drop rapidly at mitosis (Spencer et al., 2013), with the onset of cyclin A destruction, as illustrated in Figure 2A. Following mitosis, in most proliferating cells (of many different lines), CDK/E,A activity immediately begins rising again (CDK/E,A^*increasing*^ cells), increasing monotonically to a peak at the next mitosis (Figure 2A). The pattern of this increase for the majority of cells is broadly similar but it is noteworthy that individual traces differ, indicating a significant degree of asynchrony (Spencer et al., 2013). The increase is driven first by cyclin E accumulation, in conjunction with CDK2, and then by cyclin A accumulation as it replaces cyclin E, with CDK2 later supplemented by CDK1 (Spencer et al., 2013; Barr et al., 2016; Schwarz et al., 2018). However, as indicated in Figure 2A, in a significant minority of cells (typically of the order of 15–30% of the population, depending on cell type), the CDK/E,A activity fails to rise again immediately after mitosis (CDK/E,A^*low*^ cells), either remaining low for the remainder of the experiment or increasing again after a variable delay, indicating the start of another cycle (Spencer et al., 2013; Arora et al., 2017; Yang et al., 2017; Moser et al., 2018; Min et al., 2020). These CDK/E,A^*low*^ cells have hypophosphorylated RB and remain mitogen sensitive for re-entry into the cycle, indicating that they are pre-Restriction Point cells in G0 (Spencer et al., 2013; Cappell et al., 2016; Arora et al., 2017; Moser et al., 2018). The CDK/E,A^*increasing*^ cells, however, appear to be committed to the next cell cycle from birth. They already have hyperphosphorylated RB from the very start of the cycle and are insensitive to mitogen withdrawal or MAPK pathway inhibition (Spencer et al., 2013; Yang et al., 2017; Min et al., 2020). Indeed, mitogen withdrawal or MAPK pathway inhibition must be applied during the mother cell cycle to prevent or delay cell cycle re-entry in the CDK/E,A^*increasing*^ daughters (Spencer et al., 2013; Yang et al., 2017; Min et al., 2020).

This bifurcation in CDK/E,A activity occurs within what is otherwise a homogeneous population. For rapidly growing cells, it is attributable in large part to induction and variable expression of p21 (Spencer et al., 2013; Overton et al., 2014; Arora et al., 2017; Barr et al., 2017; Yang et al., 2017; Moser et al., 2018; Heldt et al., 2018). Thus, p21 levels are higher in the out-of-cycle CDK/E,A^*low*^ cells and decline as cells re-enter the cycle and switch to the CDK/E,A^*increasing*^ state. Importantly, in populations of rapidly proliferating cells, the dropping out of cycle marked by the bifurcation in CDK/E,A activity is much reduced after knockdown of p21 with siRNA or gene inactivation, supporting a causal role for p21 in cell cycle exit (Spencer et al., 2013; Overton et al., 2014; Barr et al., 2017; Heldt et al., 2018).

The induction of p21 in some cells but not others is, in turn, due to a p53-mediated DNA-damage response in the mother cell, passed on through mitosis to the daughter cells (Arora et al., 2017; Barr et al., 2017; Yang et al., 2017; Heldt et al., 2018). This DNA-damage response is most likely a result of replication stress (e.g., stalled replication forks) during S phase in the mother cell (Harrigan et al., 2011; Lukas et al., 2011; Koundrioukoff et al., 2013; Moreno et al., 2016). Such replication stress is a fairly frequent (and expected) occurrence in mammalian DNA replication, though other causes of endogenous DNA damage are also possible. Experimentally induced replication stress or DNA-damage in the mother cell cycle from treatment with low doses of aphidicolin or neocarzinostatin (insufficient to cause arrest in G2), also led to elevated levels of p21 in daughter cells and increased exit from the cycle into the CDK/E,A^*low*^ state (Arora et al., 2017; Barr et al., 2017; Yang et al., 2017).

Consistent with the involvement of a DNA damage response, cells with high levels of p21 exhibit a higher frequency of DNA-damage foci (positive for 53BP1 or γH2AX) than cells with low p21 (Arora et al., 2017; Barr et al., 2017). In turn, the greater the number of DNA-damage foci, the longer it takes for the cells to re-enter the cycle and start increasing CDK/E,A activity once more (Arora et al., 2017). Nevertheless, not all CDK/E,A^*low*^ cells have high p21 (Spencer et al., 2013) or show DNA damage foci (Arora et al., 2017) and it was suggested that the presence of foci may account for only around 50% of the cells that undergo transient arrest after mitosis (Arora et al., 2017). This raises the possibility that the transient arrest of some cells may be due to something other than a DNA-damage response and elevated p21. In this connection, it is worth noting that Hs68 human fibroblasts immortalized with hTERT, growing optimally in high serum, have cycle times ranging from around 10–12 h to more than 95 h (Nassrally et al., 2019). With S + G2 being no more than 10–12 h (the minimum cycle time), the more than 70% of cells dividing with ages greater than 22 h would have spent longer than 10 h in G1, a length taken (in some studies) as indicative of cell cycle exit (Barr et al., 2017). Nevertheless, only 10–15% of proliferating Hs68-hTERT typically show 53BP1-positive foci (Nassrally et al., 2019), indicating that the majority of slow transits through G1 in this cell type must have been due to something other than a DNA-damage response. This additional factor may be related to proliferation rate *per se*.

In the original experiments of Spencer et al. (2013) using MCF10A cells in full growth medium, roughly 25% of the cells left the cycle after each mitosis into the CDK/E,A^*low*^ state (Figure 2A). However, in later work by Overton et al. (2014) using the same cell type, there was no such cell cycle heterogeneity under high growth factor conditions (20 ng/ml EGF; 5% serum). Only when growth factor stimulation was reduced fourfold (to 5 ng/ml EGF; 1.25% serum) did cells exit the cycle after mitosis with the frequency reported by Spencer et al. (2013). Thus, in the original experiments, the cells may not have been growing at quite their maximum rate, for reasons unknown, despite being in similar high growth factor medium. This slightly reduced growth rate may have contributed to some of the cell cycle drop-out after mitosis, in addition to a DNA-damage response. The lack of any bifurcation in CDK/E,A activity in a line of MCF10A cells deleted for both alleles of p21 was taken as evidence that the drop-out was entirely dependent on p21 (Spencer et al., 2013), as noted previously. However, the p21^–/–^ MCF10A cells used had acquired a reduced dependence on EGF during their isolation (Bachman et al., 2004), compared to the parental line, and may therefore have been less sensitive to any slight deficiency in the growth conditions. A role for reduced mitogenic stimulation in the bifurcation in CDK/E,A activity, in addition to a DNA-damage response mediated by p21, cannot therefore be ruled out.

Further support for the possible importance of growth rate and the level of mitogenic stimulation, in contributing to the heterogeneity in cell exit after mitosis, comes from the results obtained with Swiss 3T3 cells. It was reported that these cells, nominally growing optimally but with an average cycle time of ∼30 h, showed a very high rate of cell cycle drop-out after mitosis, with 77% of the cells passing into the CDK/E,A^*low*^ state (Spencer et al., 2013). However, in my own laboratory, Swiss 3T3 cells in high serum grew at twice the rate, with a median cycle time of around 15 h, and showed no such cell cycle heterogeneity. Rather, cell cycle drop-out was seen only when the cells were grown in sub-optimal serum concentrations (Brooks and Riddle, 1988a,b). It seems probable therefore that some of the bifurcation in CDK/E,A activity seen with Swiss 3T3 cells (Spencer et al., 2013) may have been a consequence of a suboptimal growth rate (due, perhaps to medium composition, which was not specified), and not due solely to a stress response to DNA damage mediated by p53-p21.

Given that mitogen reduction is known to lead to the induction of p27 and that its expression is heterogeneous in quiescent populations (Coats et al., 1996; Hitomi et al., 2006), such suboptimal growth could lead to the upregulation of p27 in some cells. This would be expected to add to the contribution of p21 in driving the bifurcation in CDK/E,A activity. Clearly, live cell imaging experiments looking at both p21 and p27 simultaneously are needed to help unpick their relative importance under different conditions, in different cell types. Until this is done, a role for p27 in causing some of the transient cell cycle exit into the CDK/E,A^*low*^ state, in proliferating populations, cannot be ruled out.

## Bypass of the Restriction Point in Continuously Cycling Cells

As already discussed, in continuously cycling cells, some 15–30% of the population typically enter the CDK/E,A^*low*^ state after mitosis in which CDK/E,A activity fails to increase immediately (Figure 2A). These cells are born with hypophosphorylated RB and require mitogenic stimulation to re-enter the cycle. For these cells, the standard Restriction Point model seems appropriate. However, CDK/E,A^*increasing*^ cells, the majority, have hyperphosphorylated RB from birth and are independent of mitogenic stimulation for continued progress through the cell cycle. These cells are, it seems, already committed to the next cell cycle from birth, which calls into question the idea that the Restriction Point is a critical decision point in G1 which *all* cells must pass through before continuing to the next cycle.

The key determinant of whether cells enter the CDK/E,A^*increasing*^ or CDK/E,A^*low*^ paths appears to be the level of CDK4,6/D activity immediately after mitosis (Yang et al., 2017; Min et al., 2020). This in turn depends on the levels of cyclin D inherited from the mother cell, along with any p21/p27. Importantly, interruption of mitogenic signaling with a MEK inhibitor at any point in the mother cell cycle, even for as little as 1 h, affects the level of cyclin D attained at the end of G2 (Min et al., 2020). This is not due to a direct effect on cyclin D expression but rather to a decrease in overall translation rate which persists to the end of G2, whenever the pulse of MEK inhibitor is given earlier in the cycle. Conversely, treatment of mother cells with the CDK4,6 inhibitor palbociclib (which blocks the cell cycle but not cell growth in mass) produces enlarged cells with elevated translation capacity. These enlarged cells no longer respond to transient MEK inhibition with a reduction in the proportion of CDK/E,A^*increasing*^ cells after mitosis (Min et al., 2020). That Cyclin D is the critical aspect of translation capacity was shown by knockdown of all three cyclin D genes in mother cells with siRNA, leading to a reduced proportion of CDK/E,A^*increasing*^ daughter cells. Conversely, overexpression of cyclin D1 in mother cells, increased the proportion of CDK/E,A^*increasing*^ daughter cells (Min et al., 2020).

These findings provide good evidence for the importance of cyclin D levels and CDK4/D activity in driving the bifurcation in CDK/E,A activity after mitosis. They also offer insight into two other fundamental features of the vertebrate cell cycle. Firstly, since sister cells would inherit identical concentrations of cyclin D from the mother cell, along with any p21 or p27, this would lead to similar cell cycle trajectories immediately afterward, potentially explaining much of the well-known correlation in sibling cycle times (Minor and Smith, 1974; Brooks et al., 1980). Secondly, since the level of cyclin D at the end of G2 is related to overall translation rate, large cells, with a high translational capacity, would generate large daughter cells with high cyclin D. These in turn would be expected to have shorter than average G1 times, providing a possible explanation for at least some of the observed inverse correlation between cell size and G1 duration (Shields et al., 1978; Ginzberg et al., 2018; Min et al., 2020; Zatulovskiy and Skotheim, 2020).

## The Relative Contributions of CDK4,6/Cyclin D and CDK2/Cyclin E in RB Hyperphosphorylation

According to the standard model of the Restriction Point (Planas-Silva and Weinberg, 1997; Pennycook and Barr, 2020), CDK4,6/Cyclin D initiates phosphorylation of RB sufficient to partially derepress E2F, leading to the expression of cyclin E. This in turn activates CDK2 to fully phosphorylate (hyperphosphorylate) RB, promoting further expression of cyclin E and setting up the positive feedback loop essential to the bistability of the RB-E2F switch (Figure 1). An ingrained notion in this scheme is that CDK4,6/D is insufficient on its own for full phosphorylation of RB but instead requires help from CDK2/E, which eventually replaces it altogether. These ideas are challenged by the observation that CDK/E,A^*increasing*^ cells are already mitogen independent and have fully phosphorylated RB from birth, when CDK/E activity is at its lowest (Spencer et al., 2013; Moser et al., 2018; Chung et al., 2019). Moreover, treatment with the CDK4,6 inhibitor palbociclib at any point in G1, right up to shortly before entry into S phase, reverses RB-hyperphosphorylation rapidly and completely (Chung et al., 2019). This is true not only of the CDK/E,A^*increasing*^ cells in cycling populations, but also of cells stimulated from quiescence that had become mitogen independent (i.e., post-Restriction Point) several hours earlier (Chung et al., 2019). Only after entry into S phase is RB hyperphosphorylation independent of CDK4,6/D (insensitive to palbociclib) and instead dependent solely on CDK/E,A activity (and sensitive to a CDK2 inhibitor) (Chung et al., 2019).

On the face of it, these observations suggest that there is no role at all for CDK2/E activity in RB hyperphosphorylation throughout G1, calling into question the validity of the RB-E2F bistable switch as the basis for the Restriction Point (Yao et al., 2008). However, the findings of Chung et al. (2019) are in apparent conflict with compelling evidence from Narasimha et al. (2014) that CDK4,6/cyclin D is only able to *mono*phosphorylate RB and that hyperphosphorylation does not occur until after the appearance of CDK2/Cyclin E activity in “late” G1. These studies made use of isoelectric focusing to separate unphosphorylated RB from isoforms phosphorylated on 1, 2, 3,…, up to 14 sites. Mitogen-stimulated human fibroblasts and other cell types had only monophosphorylated RB until an abrupt shift to fully phosphorylated RB (on 14 sites) coincident with the appearance of CDK2/Cyclin E activity in what was described as “late” G1 (though the timing of DNA synthesis was not shown). The kinase responsible for the RB monophosphorylation was confirmed as CDK4,6/cyclin D by its absence in Cyclin D triple-knockout mouse embryo fibroblasts (lacking all three cyclin D genes) and by its sensitivity to palbociclib and p16 (which disrupts CDK4,6-cyclin D complexes), whereas the hyperphosphorylation was sensitive to a CDK2-inhibitor. Remarkably, the monophosphorylated RB was phosphorylated on any one, but *only* one, of 14 different sites. The different monophosphorylated RB isoforms differed slightly in their affinity for different E2F family members, but all appeared active in suppressing E2F function. This raises the question as to how cyclin E is ever switched on, in order to trigger CDK2/Cyclin E-mediated RB-hyperphosphorylation, leading Narasimha et al. (2014) to invoke the existence of some other mechanism for inducing cyclin E expression not involving E2F.

The contrary conclusions of Chung et al. (2019) that CDK4,6/D alone is sufficient for RB hyperphosphorylation depend heavily on the use of palbociclib to inhibit CDK4,6/D activity. However, recent studies of the crystal structure of trimeric complexes between CDK4, cyclin D1 and either p21 or p27 indicate that the action of palbociclib is much more nuanced than previously appreciated (Guiley et al., 2019; Sherr, 2019). Although p21 and p27 are strong inhibitors of CDK1,2/E,A activity, their action on CDK4,6/D has been less clear-cut. In contrast to other CDK-cyclin complexes, CDK4,6 associates poorly with cyclin D, requiring the assistance of assembly factors, which include Hsp90, Cdc37 and p21 or p27. Indeed, mouse embryo fibroblasts lacking both p21 and p27 are unable to form active CDK4-Cyclin D complexes (Cheng et al., 1999). The structural studies of Guiley et al. showed how p21 and p27 are able to bring CDK4 and Cyclin D together, facilitating complex formation and promoting conformational changes conducive to enzymatic activity (Guiley et al., 2019). Nevertheless, the p21-CDK4-Cyclin D1 trimer lacked kinase activity, making p21 an inhibitor, consistent with single-cell imaging data from Yang et al. (2017). (This, of course, implies that p21 would need to dissociate from the CDK4-cyclin D dimer after facilitating assembly if it is to function as a promoter of activity.) As with p21, the binding of p27 was also inhibitory until, that is, it is phosphorylated on Tyr74 (a site lacking in p21). Assayed *in vitro*, the resulting tyrosine-phosphorylated p27-CDK4-Cyclin D1 trimer was an active kinase for RB and other CDK4/D targets such as CDC6. Moreover, the tyrosine-phosphorylated trimer had a lower Km for ATP than the CDK4-Cyclin D1 dimer (0.4 mM vs. 1.5 mM), leading to the suggestion that tyrosine-phosphorylated p27 is an allosteric *activator* of CDK4-Cyclin D1 (Guiley et al., 2019). However, since ATP levels inside cells are typically in the mM range, the change in Km is unlikely to have any major impact on physiological activity. More significant was the finding that the kinase activity of the tyrosine-phosphorylated trimer was not inhibited by palbociclib at all (Guiley et al., 2019), in contrast to the strong inhibition of the CDK4-Cyclin D1 dimer. Indeed, the binding of p27 and palbociclib to Cdk4 were found to be mutually exclusive. Furthermore, in cells arrested in G1 after prolonged treatment with palbociclib (for 48 h), levels of RB kinase activity in Cdk4 or cyclin D1 immunoprecipitates remained unchanged. Instead, there was a reduction in the RB kinase activity associated with CDK2 due, at least in part, to increased levels of p21 in the immunoprecipitates. Guiley et al. concluded that the cell cycle arrest induced by palbociclib was due to an indirect inhibition of CDK2, possibly through disrupting the p21-CDK4-Cyclin D1 trimer, leading to an accumulation of CDK4 monomer bound to palbociclib, and freeing the otherwise sequestered p21 to inhibit CDK2 (Guiley et al., 2019).

**FIGURE 3:**
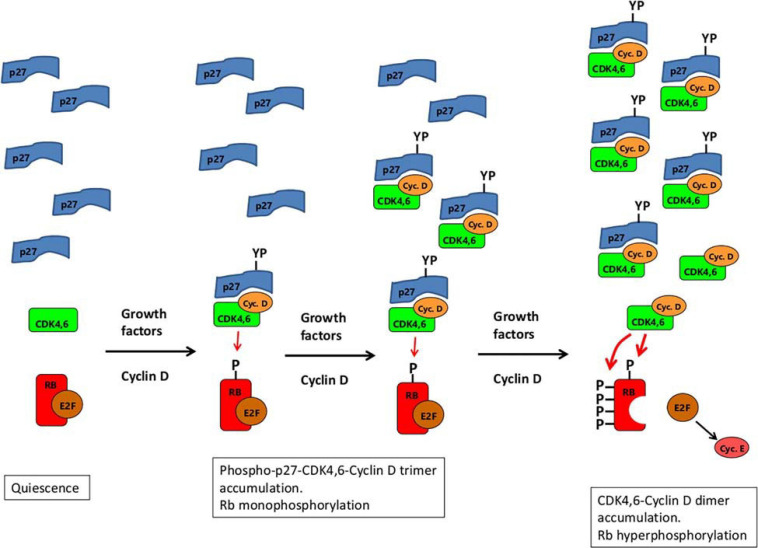
A schematic illustrating a possible explanation of both RB-monophosphorylation and RB-hyperphosphorylation by CDK4,6/cyclin D. Growth factor stimulation of quiescent cells leads the expression of Cyclin D, which complexes with CDK4,6 and p27. The trimer, when phosphorylated on Tyr74 of p27 (marked YP) is a weak kinase for RB (resistant to palbociclib), enabling monophosphorylation of RB. (Whether tyrosine phosphorylation of p27 is restricted to the trimer or applies equally to the monomer, is not known. The lack of p27 monomers marked with YP in the diagram should not be taken therefore to imply that phosphorylation of the monomer does not occur.) As cyclin D expression continues, more and more of the trimer accumulates, eventually sequestering all the p27 monomers. From then on, further production of cyclin D leads to fully active CDK4,6/cyclin D dimers (sensitive to palbociclib) that hyperphosphorylate Rb. The derepressed E2F then induces expression of cyclin E which, together with CDK2, sustains Rb hyperphosphorylation thereafter.

Although the tyrosine-phosphorylated p27-CDK4-Cyclin D1 trimer was said to be an active RB kinase, this depends on how kinase activity is measured. Using a recombinant fragment of RB consisting of amino acids 771–874, the trimer was just as active as the CDK4-Cyclin D1 dimer. However, this RB fragment lacks a C-terminal alpha helix (amino acids 892–912) that provides an essential docking site required for efficient binding to CDK4,6-Cyclin D (Topacio et al., 2019). Using a larger RB fragment (amino acids 771–928) containing this docking site, the tyrosine-phosphorylated p27-CDK4-Cyclin D1 trimer is a *far less* active kinase than the CDK4-Cyclin D1 dimer, with a 13-fold lower Vmax (Guiley et al., 2019). Thus, far from being an activator, p27 is an inhibitor of kinase activity for RB-substrates with an intact C-terminal tail. This could potentially explain the monophosphorylation of RB (Figure 3). On mitogenic stimulation of quiescent cells, the first cyclin D to be produced will be complexed with both CDK4,6 and p27 (Figure 3). After phosphorylation of p27 on Tyr74 (most likely by Src-like kinases, themselves responsive to mitogenic stimulation – Chu et al., 2007), the trimeric complex would gain sufficient kinase activity to be able to start the phosphorylation of RB (Figure 3). However, after adding the first phosphate, the association between RB and the trimer could conceivably be weakened (perhaps due to altered electrostatic charge), encouraging dissociation. Even without such weakening, by not being able to hold on tightly to the C-terminal docking helix due to blocking by p27 (Guiley et al., 2019), the p27-CDK4,6-cyclin D1 trimer might dissociate from RB before the addition of any further phosphates to it. If so, then only after sufficient cyclin D has been produced to sequester all the p27 in the cell, will CDK4,6/cyclin D dimer start to appear with full activity toward RB to ensure its hyperphosphorylation (Figure 3). (This built-in delay could be of value in enabling the cell to grow in mass before reaching a point of commitment to the next cell cycle.) Note that a possible argument against this scenario – that RB monophosphorylation was reported to be blocked by palbociclib (Narasimha et al., 2014) – can be dismissed because the palbociclib appears to have been added at the time of serum step-up, before the induction of cyclin D. As a result, the palbociclib would have bound first to CDK4,6 monomer (Guiley et al., 2019), preventing its subsequent association with cyclin D and p27.

If these speculations are correct, then the RB hyperphosphorylation seen in CDK/E,A^*increasing*^ cells immediately after mitosis could conceivably be due entirely to palbociclib-sensitive CDK4,6/cyclin D dimers, even though these may be a minor fraction of the total CDK4,6/cyclin D in the cell (Guiley et al., 2019). Nevertheless, when nearing the end of G1, cells have elevated levels of CDK2/E activity, just below those needed for entry into S phase. On inhibiting fully active CDK4,6/cyclin D dimers with palbociclib, there should be sufficient CDK2/E activity remaining to sustain RB hyperphosphorylation in the absence of CDK4,6/cyclin D activity. Instead, Chung et al. (2019) found complete reversal of RB hyperphosphorylation after just 15 min treatment with palbociclib. This implies that the CDK2/E present has no activity toward RB, which is difficult to understand. The most likely explanation would seem to be that palbociclib causes rapid dissociation of CDK4-Cyclin D1 trimer complexes with p27 or p21, freeing the sequestered p21/p27 to inhibit the CDK2/E, i.e., an indirect inhibition of CDK2 activity by palbociclib, as suggested by Guiley et al. (2019). Consistent with this, the activity of CDK2/E is seen to start falling immediately after the addition of palbociclib (Chung et al., 2019). Thus, the provocative suggestion of Chung et al. (2019) that Cyclin E plays no role in RB hyperphosphorylation until after the G1/S transition remains to be established, by ruling out any indirect inhibition of CDK2/E by palbociclib. This could perhaps be done by repeating the experiments with cells deficient for both p21 and p27. In this case, palbociclib should be unable to reverse RB hyperphosphorylation and block entry into S phase after the point of mitogen independence (passage of the Restriction Point), as there would be no sequestered p21 or p27 to relocate to and inhibit CDK2/E. However, cells lacking both p21 and p27 may be compromised in their assembly of active CKD4,6/cyclin D complexes (Cheng et al., 1999), which would complicate the experiments.

## APC/C^CDH1^ Inactivation

In continuous cycling cells or in cells stimulated from quiescence, there comes a point in G1 when CDK2/E,A activity starts to rise inexorably and monotonically, indicating the start of the next cycle (Figure 2A). Activity reaches a peak at mitosis, after which it declines rapidly toward a baseline. As the level rises from baseline to peak, a threshold is reached when the cell becomes irreversibly committed to entry into S phase (Spencer et al., 2013; Barr et al., 2016; Schwarz et al., 2018; Chung et al., 2019). A similar requirement to achieve a threshold level of CDK activity for entry into S phase is also seen in Fission yeast (Coudreuse and Nurse, 2010), suggesting that this is a universal requirement. An important question that follows on from this is how a gradually increasing level of CDK activity is translated into an abrupt, all-or-none commitment to enter S phase. Recent live-cell imaging studies suggest that inactivation of the Anaphase Promoting Complex or Cyclosome (APC/C), a multimeric ubiquitin E3-ligase, may be key to this.

The activity of APC/C depends on two substrate-recognition adaptor proteins, CDC20 and CDH1 (reviewed, Peters, 2006). Anaphase is brought about by APC/C complexed with CDC20. On exit from mitosis, CDC20 dissociates from APC/C and is replaced by CDH1, maintaining APC/C activity throughout G1. This prevents the accumulation of many proteins needed for S phase (see later). Switching off APC/C^CDH1^ is therefore essential for entry into S phase and in keeping with this, CDH1 knockdown accelerates the G1/S transition (Sigl et al., 2009; Yuan et al., 2014).

By using a fluorescent reporter construct consisting of a fragment of geminin (a well-established APC/C target) conjugated to mCherry (Sakaue-Sawano et al., 2008), it has been possible to follow the activity of APC/C^CDH1^ in living cells, in real time (Cappell et al., 2016). Remarkably, APC/C^CDH1^ activity was found to disappear extremely abruptly in a switch-like manner (as illustrated in Figure 2B), over a span of about an hour (Cappell et al., 2016). The inactivation of APC/C^CDH1^ occurred at different times in different cells (Figure 2B), but was always just before the onset of S phase. For cells stimulated from quiescence, this was several hours after RB hyperphosphorylation and passage of the Restriction Point (apparent mitogen independence). The inactivation of APC/C^CDH1^ required CDK2/E activity and was blocked by a low dose of a CDK1,2 inhibitor or by knockdown of cyclin E with siRNA (though not cyclin A). However, once inactivated (“off”), APC/C^CDH1^ could not be switched on again with the same low dose of CDK1,2 inhibitor (Cappell et al., 2016, 2018), consistent with inactivation being an irreversible transition. For the cells in which CDK/E,A activity does not rise immediately after M (the CDK/E,A^*low*^ population), APC/C^CDH1^ activity remains “on” for as long as CDK/E,A activity continues to be low. However, once CDK/E,A activity starts to rise, APC/C^CDH1^ inactivation typically follows 3–5 h later, suggesting a requirement to reach a threshold level of activity.

**FIGURE 4:**
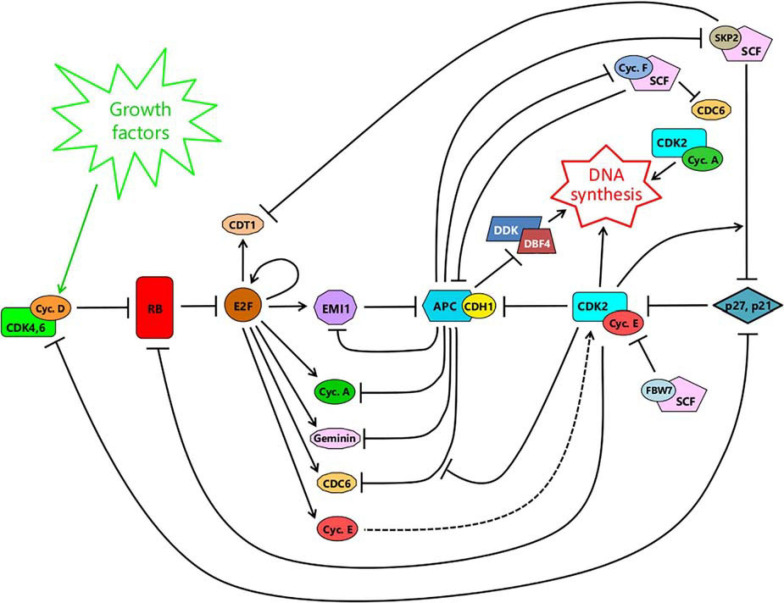
A schematic integrating the RB-E2F switch **(left)** with the APC/C^CDH1^ switch **(center-right)**. For details, see text.

A schematic illustrating the major interacting networks surrounding APC/C^CDH1^ and its connections to the RB-E2F switch and the onset of DNA synthesis, is shown in Figure 4. The abruptness of the fall in APC/C^CDH1^ activity, once it starts (Figure 2B), is unaffected by a pan-cullin inhibitor (Cappell et al., 2016). This indicates that the sudden, sharp fall in APC/C^CDH1^ activity is not dependent on the SCF (Skp1/CUL1/Fbox protein) family of cullin E3 ubiquitin ligases that become active from late G1 to the end of G2, following APC/C^CDH1^ inactivation (Figure 4). In keeping with this, knockdown of the SCF-substrate adaptor Cyclin F, with siRNA, also failed to alter the kinetics of APC/C^CDH1^ inactivation (Cappell et al., 2018), even though SCF^*cyclin F*^ is able to target CDH1 for destruction, and vice versa (Choudhury et al., 2016). Thus, the potential double-negative feedback loop between APC/C^CDH1^ and SCF^*cyclin F*^ (Choudhury et al., 2016) – see Figure 4 – does not appear to be involved in controlling the abruptness of APC/C^CDH1^ inactivation (Cappell et al., 2018), though it could contribute to its timing or the maintenance of inactivation once it has occurred. Likewise, knockdown of another SCF-substrate adaptor, SKP2, with siRNA, also fails to alter the kinetics of APC/C^CDH1^ inactivation (Cappell et al., 2018). This shows that the abruptness of inactivation is not the result of increased CDK2/E activity following SCF^*SKP*2^-mediated elimination of p21 or p27 from inhibitory complexes with CDK2/E (Barr et al., 2016, 2017), though again some contribution to timing or maintenance of the APC/C^CDH1^ switch cannot be ruled out (Figure 4). In contrast, siRNA-mediated knockdown of the APC/C^CDH1^ inhibitor EMI1 *does* slow the rate of APC/C^CDH1^ inactivation (Cappell et al., 2016, 2018). Moreover, although inactivation of APC/C^CDH1^ still occurs after elimination of EMI1, after a delay during which CDK/E activity continues to rise, it is no longer irreversible to treatment with a low dose of CDK1,2 inhibitor. In addition, overexpression of EMI1 brought forward the abrupt inactivation of APC/C^CDH1^ (Cappell et al., 2016). Thus EMI1 controls the timing, abruptness and irreversibility of APC/C^CDH1^ suggesting that it plays a key part in the bistability of the switch (Figure 4). This bistability arises because EMI1 is both a substrate of APC/C^CDH1^ at low concentrations, and an inhibitor of it at high concentrations, creating a double-negative feedback loop (Cappell et al., 2018).

Expression of EMI1 is induced by E2F (Hsu et al., 2002) after RB hyperphosphorylation and the RB-E2F switch (Figure 4). Initially, the newly made EMI1 is targeted for destruction by APC/C^CDH1^, keeping its concentration low. However, as CDK2/E activity rises, it phosphorylates and gradually inhibits APC/C^CDH1^ allowing EMI1 to evade destruction and start accumulating. On reaching a threshold, EMI1 switches from being a substrate to being an inhibitor, further suppressing APC/C^CDH1^. This favors yet further accumulation of EMI1 culminating in complete inactivation of APC/C^CDH1^. Thus, the threshold for this switch is governed by both the level of CDK2/E activity and the concentration of EMI1. Without EMI1, inactivation of APC/C^CDH1^ can still occur but requires a much higher CDK2/E activity, thereby delaying the switch. Conversely, upregulation of EMI1 brings forward the switch (Cappell et al., 2016, 2018).

## APC/C^CDH1^ Inactivation Marks the Point of No Return in the Cell Cycle

Passage of the Restriction Point – usually equated with flipping of the RB/E2F switch and the acquisition of mitogen independence – is widely considered to be the critical point of no return in the cell cycle, when cells become irreversibly committed to the next cycle. However, the fact that entry into S phase can be blocked by palbociclib almost right up to the G1/S transition, with reversal of RB hyperphosphorylation, calls this into question (Chung et al., 2019). Regardless of the precise mechanism of action of palbociclib (as discussed earlier), this indicates a continuing role for CDK4,6/D (either direct, through RB phosphorylation, or indirect, through sequestering p27 or p21 away from CDK2/E) almost right up to the start of DNA synthesis. In contrast, once APC/C^CDH1^ is inactivated, palbociclib is no longer able to arrest the cycle, consistent with inactivation being an irreversible transition.

In addition to treatment with palbociclib, cellular stress has also been reported to reverse passage of the Restriction Point, returning cells to quiescence (Cappell et al., 2016). When “early” G1 cells were exposed to a low dose of neocarzinostatin (NCS) to induce DNA damage, at a time when RB was already hyperphosphorylated and CDK/E,A activity had started to rise, some of the cells transiently reverted to having unphosphorylated/monophosphorylated RB, and the rise in CDK/E,A activity was delayed (for around 8 h). In addition, APC/C^CDH1^ activity remained “on.” Similar results were seen after hypertonic treatment or exposure to hydrogen peroxide. If, after treatment with NCS, the cells were deprived of mitogens, most of those that reverted to unphosphorylated/monophosphorylated RB remained in this state, maintaining low CDK/E,A activity, suggesting a return to quiescence and the regaining of mitogen dependence. Consistent with this, when mitogens were restored, the cells resumed cycling, with CDK/E,A activity rising once more, followed by APC/C^CDH1^ inactivation. However, the lag between mitogen restoration and the rise in CDK/E,A activity or APC/C^CDH1^ loss was very short (less than 4 h, though rather imprecise) – much less than the normal pre-replicative lag (of around 12 h). Evidently, stress exposure had not returned the cells to a normal quiescent (G0) state. Nevertheless, the same stress exposures given after APC/C^CDH1^ had switched off were without effect, and did not lead to APC/C^CDH1^ reactivation. This reinforces the conclusion that once APC/C^CDH1^ has been inactivated, the cell passes into a state from which it cannot easily return.

One of the few interventions known to allow reactivation of APC/C^CDH1^ once it is switched off involves the knockdown of EMI1 (Barr et al., 2016; Cappell et al., 2016, 2018). However, such reactivation, after entry into S phase, leads to re-replication of DNA (Barr et al., 2016), so is not without deleterious consequences for the cell.

The only other intervention known to induce APC/C^CDH1^ reactivation, after it has been switched off, is DNA damage (Wiebusch and Hagemeier, 2010; Segeren et al., 2020). On the face of it, this might seem to contradict the findings of Cappell et al. (2016), who found no such reactivation, as indicated above. However, the time span of these studies is quite different. Starting with cells that had just undergone APC/C^CDH1^ inactivation, Cappell et al. (2016) found no reactivation, following a short pulse with a low dose of NCS, over the next 8 h. In the studies by Wiebusch and Hagemeier (2010) and Segeren et al. (2020), somewhat higher levels of DNA damaging agents also produced little change in APC/C^CDH1^ activity over the first 6 h. Only *after* this did APC/C^CDH1^ levels start to rise, reaching a maximum roughly 12 h later. This reactivation of APC/C^CDH1^ was accompanied by the loss of EMI1, through degradation and the suppression of transcription (Wiebusch and Hagemeier, 2010). Importantly, experimental upregulation of EMI1 in S phase through knock-out of the E2F repressors E2F7/8, or overexpression of an activator E2F (E2F3), prevented the reactivation of APC/C^CDH1^ (Segeren et al., 2020) after DNA damage, again underscoring the importance of EMI1 in maintaining the APC/C^CDH1^ off-state (c.f. Figure 4). The pathway through which DNA damage brings about APC/C^CDH1^ reactivation involves the induction of p53, which in turn acts partly through transactivation of E2F7 (and suppression of EMI1), but in particular through upregulation of p21 (Wiebusch and Hagemeier, 2010; Segeren et al., 2020). Exactly how p21 contributes to the reactivation of APC/C^CDH1^ was not established, but it is likely to be through inhibition of CDK2/cyclin E (c.f. Figure 4). Previously, Cappell et al. (2018) had reported no reactivation of APC/C^CDH1^ once it had switched off, using the maximum tolerated dose of a small molecule CDK1,2 inhibitor. However, the very high levels of p21 seen after DNA damage (Wiebusch and Hagemeier, 2010; Segeren et al., 2020) may have achieved a more-complete inhibition of CDK2/cyclin E, sufficient to reverse the switch (Cappell et al., 2018). As with EMI1 knock-down above (Barr et al., 2016), the consequences of this premature reactivation of APC/C^CDH1^, after DNA damage, are not benign, and include cell senescence (Wiebusch and Hagemeier, 2010) or re-replication (Segeren et al., 2020). Clearly, inappropriate reversal of the APC/C^CDH1^ switch is not something easily tolerated.

## APC/C^CDH1^ Inactivation Represents a Change of State in the Cell Cycle

**FIGURE 5:**
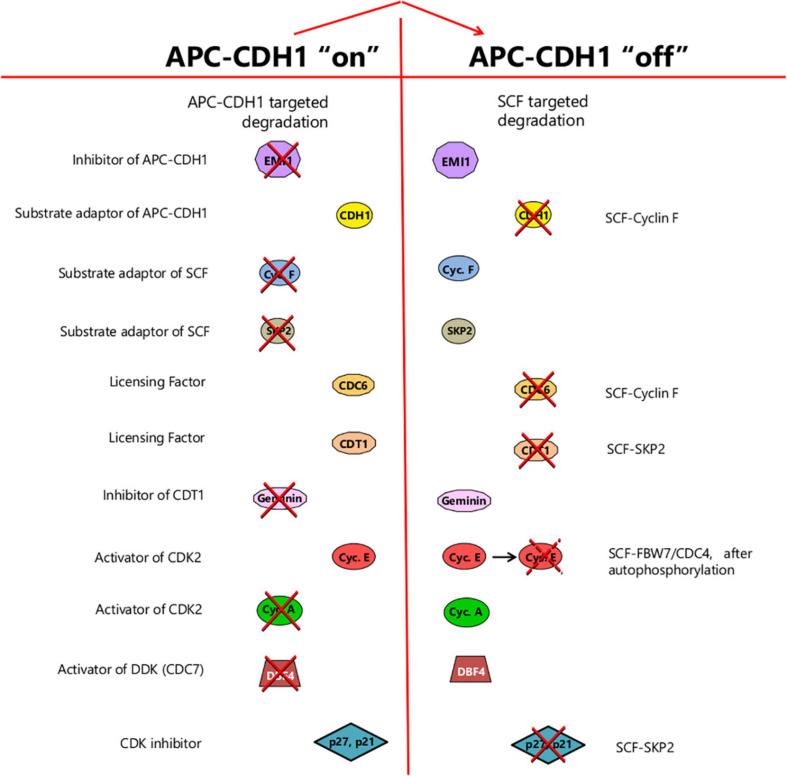
The change of state resulting from APC/C^CDH1^ inactivation. Prior to this transition, the cell is in a state where proteolysis targeted by APC/C^CDH1^ predominates, leading to the elimination of EMI1, its own inhibitor, together with substrate adaptors for the SCF-ubiquitin ligase, and components needed for DNA synthesis such as DBF4 and cyclin A. Factors required for replication origin licensing (CDC6 and CDT1) are stable whereas geminin – an inhibitor of CDT1 and hence licensing – is destroyed. Following the switch, SCF-mediated-proteolysis is promoted by the stabilization of its substrate adaptors (such as SKP2 and cyclin F). Origin firing becomes possible on accumulation of DBF4, acting together with CDK2 activity promoted first by cyclin E, then cyclin A, with activity further enhanced by p27 and/or p21 elimination. Origin relicensing is prevented in part by SCF-targeted degradation of the CDC6 and CDT1 licensing factors together with CDT1 suppression through geminin accumulation. Thus, at the point of APC^CDH1^ inactivation, the cell passes from a state where origin licensing is possible but origin-firing is prevented, to one where origin-firing becomes possible but relicensing is suppressed. The change of state, once it occurs, is irreversible due to EMI1 stabilization and SCF-mediated elimination of CDH1.

Following the sudden inactivation of APC/C^CDH1^, ubiquitin-mediated proteolysis in the cell cycle shifts abruptly from being APC/C^CDH1^-dependent to primarily SCF-dependent, marking a fundamental change of state (Figure 5). Prior to this transition, CDH1 is stable, targeting APC/C to the destruction of its own inhibitor, EMI1 (as already discussed). Also targeted are Cyclin F (Choudhury et al., 2016) and SKP2 (Bashir et al., 2004; Wei et al., 2004), substrate adaptors for SCF, keeping them “switched off.” After the transition switching off APC/C^CDH1^, EMI1 is stabilized and maintains the suppression of APC/C^CDH1^. Since cyclin F is no longer eliminated, SCF^*cyclin F*^ also now accumulates, targeting CDH1 for proteolysis and further reinforcing the irreversibility of APC/C^CDH1^ inactivation (Choudhury et al., 2016). Similarly, stabilization of SKP2 after APC/C^CDH1^ inactivation leads to the formation of SCF^*SKP*2^, which targets any p21 or p27 present for proteolysis, after they have been phosphorylated by CDK2/E (Lu and Hunter, 2010). The active CDK2/E released would in turn further assist in maintaining the suppression of APC/C^CDH1^ (Figure 4). Indeed, since phosphorylation of SKP2 on Ser64 by CDK2/E can prevent its interaction with APC/C^CDH1^ (Rodier et al., 2008), stabilizing it, it is possible that some SCF^*SKP*2^ may appear even before full APC/C^CDH1^ inactivation, contributing to the timing of the switch, through p21 or p27 elimination and CDK2/E activation (Barr et al., 2016, 2017; Heldt et al., 2018).

Prior to the APC/C^CDH1^ “off” transition, factors required for DNA synthesis are actively destroyed. These include Cyclin A, which maintains CDK2 activity after the decline of Cyclin E following the start of S phase, and DBF4, an activator of CDC7 kinase, also known as DDK (DBF4-dependent kinase) (Figure 4). Conversely, the licensing of replication origins is favored. Licensing involves recruiting the MCM helicase to origins of replication, mediated by the licensing factors CDT1 and CDC6. These in turn bind to the hexameric Origin Recognition Complex (ORC1-6) assembled at potential initiation sites along the DNA (Blow and Dutta, 2005; Fragkos et al., 2015). Expression of both CDT1 and CDC6 is induced by E2F (Figure 4). However, CDC6 is initially targeted for destruction by APC/C^CDH1^ until it gets phosphorylated by CDK2/cyclin E, which blocks recognition by CDH1, stabilizing it (Mailand and Diffley, 2005; Figure 4). This opens up a window for origin licensing between the appearance of CDK2/cyclin E and the inactivation of APC/C^CDH1^.

After the inactivation of APC/C^CDH1^, DBF4 and cyclin A are no longer targeted for proteolysis and are able to accumulate, facilitating, along with CDK2 and cyclin E, the initiation of DNA synthesis (Fragkos et al., 2015). At the same time, CDC6 and CDT1 become targeted for destruction by SCF^*cyclinF*^ and SCF^*SKP*2^ respectively (Figures 4, 5), terminating the capacity for further origin licensing (or re-licensing). In addition, geminin (an inhibitor of CDT1 through direct binding), is now able to accumulate (Figures 4, 5), reinforcing the suppression of origin licensing. Thus, after APC/C^CDH1^ inactivation, cells pass from a state where origin licensing is possible but DNA replication is not, to one where the initiation of DNA synthesis can occur but relicensing cannot.

## The G1/S Transition and the Initiation of DNA Synthesis

Entry into S phase, marked by the initiation of DNA synthesis, occurs with inevitability and very quickly (in an hour or so), after APC/C^CDH1^ is switched off (Cappell et al., 2016). When DNA synthesis begins in a given cell, it does so simultaneously at dozens of separate replication foci scattered throughout the nucleus (Newport, 1996). This would seem to suggest a sudden, global change of state acting throughout the nucleus to trigger the onset of DNA synthesis at multiple origins, but what this might be remains unclear. The firing of replication origins requires both CDK activity (driven by either cyclin E or cyclin A) and DDK activity (driven by DBF4) (Fragkos et al., 2015). As discussed earlier, CDK/E,A activity increases rather gradually during G1 and it is difficult to see how just attaining a threshold could have the switch-like precision to trigger the synchronous firing of numerous, dispersed replication foci, each consisting of multiple replication origins. The requirement for DDK activity explains why the initiation of DNA synthesis cannot take place before APC/C^CDH1^ is switched off since it is only after this that DBF4 is able to accumulate (Figure 5). However, the gradual accumulation of DBF4, after the switch, again makes it difficult to explain simultaneous initiation at multiple foci, at the start of S phase. The fact that it requires *both* CDK and DDK activity adds an element of cooperativity, but synchronous origin firing remains difficult to understand in the absence of a switch-like mechanism acting throughout the nucleus.

Although the number of licensed origins declines with time during quiescence, in recently quiescent cells enough origins remain licensed for the isolated (intact) nuclei to enter DNA synthesis when transferred into cytoplasmic S phase extracts from Xenopus eggs (Sun et al., 2000). Nevertheless, individual nuclei apparently begin DNA synthesis at different times, despite being present in the same S phase cytoplasm (Hola et al., 1994, 1996). The asynchrony is not correlated with differences in nuclear transport (Sun et al., 2001) and is therefore unlikely to be caused by differences between nuclei in the time taken to accumulate replication factors to a critical threshold. Instead, individual quiescent nuclei must vary in their sensitivity to the replication-inducing factors in Xenopus egg extracts. Remarkably, when permeabilized binucleate cells are added to such extracts, *both* nuclei of a pair show identical levels of DNA synthesis suggesting synchrony in the timing of initiation, even though different pairs of nuclei start DNA synthesis at different times (Hola et al., 1996). Clearly, whatever determines the sensitivity of nuclei to the inducers of DNA synthesis, it is a property shared by nuclei formed in a common cytoplasm. (The binucleate cells were generated by blocking cytokinesis with cytochalasin B, prior to serum withdrawal to render them quiescent.) One possibility that might explain this is the degree of chromatin condensation. It is well known that different sets of replication origins fire at different (and reproducible) times throughout S phase, with heterochromatin (the most-condensed) replicating late (Fragkos et al., 2015). Thus the differences between pairs of binucleates in the time taken to start replicating may simply reflect the degree of chromatin compaction at the time of permeabilization and exposure to egg cytoplasm. That such chromatin differences exist may be inferred from the studies of Yen and Pardee (1979), who noted for quiescent 3T3 cells that nuclear volume varied over a twofold range, despite DNA content being the same. Moreover, the cells with the largest nuclei (and, presumably, the most dispersed chromatin) were the first to start DNA synthesis after serum stimulation. Thus, the first nuclei to start DNA synthesis in egg extracts may simply be the ones with the most-open chromatin. This in turn offers a plausible explanation for the apparently simultaneous firing at multiple replication foci scattered throughout the nucleus on entry into S phase. These clusters of early firing origins are simply those in the most open chromatin, able to respond to the lowest level of replication-inducers as the cell first enters S phase, with origins in more-compacted chromatin firing later as the concentration of inducers in the nucleus rises, or the chromatin becomes decondensed. The apparently synchronous firing of multiple foci at the start of S phase may therefore be a reflection merely of similar origin-sensitivity (accessibility), rather than the response to some global change of state extending throughout the nucleus at the time of the G1/S transition, downstream of APC/C^CDH1^ inactivation. If so, then the interval between the APC/C^CDH1^ “off” transition and the start of DNA synthesis, though short, would be expected to show some variation, inversely with nuclear volume (a surrogate for chromatin compaction). However, any such variance would be expected to be shared by sister cells, as with the nuclei of binucleates.

## Bistable Switches and Random Transitions in the Cell Cycle

For cells stimulated to re-enter the cell cycle from quiescence, the APC/C^CDH1^ inactivation switch is triggered by the rise in CDK2/E activity that follows on from passage of the Restriction Point, which in turn is triggered by RB-hyperphosphorylation and the RB-E2F switch. It seems reasonable to ask whether these two bistable switches might correspond to the two random transitions postulated in the revised Transition Probability model (Brooks et al., 1980).

The original version of the Transition Probability model (Smith and Martin, 1973) proposed that cell cycle commitment involved a single random transition in G1, the probability of which varied with conditions. The transition, if it existed, divided the cycle into two parts, an A state in G1 in which cells “waited” for commitment to occur, and a deterministic B phase (the rest of the cell cycle, including S, G2, M and part of G1) which cells were obliged to complete, once started. The model accounted for the connection between cell cycle variability and the regulation of proliferation, for the exponential tail of the distribution of cycle times and for various other aspects of cell cycle kinetics including the invariable difficulty of synchronizing cells from one cycle to the next. Nevertheless, at first sight the model seemed unlikely since it was known that the cycle times of sister cells were highly correlated, with correlation coefficients typically of the order of 0.5. If each cycle were initiated purely at random then there is no reason why sister cell cycle times should be any more alike than random pairs. Nevertheless, sister cells rarely divide at exactly the same time and it transpired that the distribution of *differences* between sister cell cycle times (the so-called β-curve) was a near perfect exponential, indicating that all of the differences could be accounted for solely in terms of a single random transition (Minor and Smith, 1974; Shields, 1977, 1978; Shields and Smith, 1977). Cycle times as a whole, however, are more variable than predicted by a single random transition. It follows that B phase must vary in general, while being identical in sister cells.

The Two Transition extension of the Smith and Martin model (Brooks et al., 1980; Brooks, 1981) arose from attempts to account for the lag preceding entry into S phase following the restoration of serum to serum-starved, quiescent cells. After the lag, cells entered S phase with what appeared to be first order kinetics, as discussed earlier, consistent with the Smith and Martin model (Brooks, 1975, 1976). The random transition – if there was one – had to be placed toward the end of the lag, since this was always much longer than the minimum G1 of continuously cycling cells. Consistent with this, the rate of entry into S phase declines quite quickly after removing the serum once more (Brooks, 1976). To explain the lag, and its independence on the level of stimulation, it was proposed that some lengthy process or processes, taking up most of the lag (and therefore called L) had to be completed before entry into S phase became possible. Process L was considered to start stochastically (the so-called Q transition), with a probability dependent on the level of stimulation. On completion of L, the cell was competent to undergo commitment to enter S phase, this corresponding to the Smith and Martin transition. At the same time, the “clock” was reset so that L could begin again (stochastically) in readiness for the next cell cycle. However, since L was longer than S + G2, it would be completed after mitosis in G1 of the daughter cells. Accordingly, sister cells would reach the point of commitment to S phase (the Smith and Martin transition) at the same time, thereby explaining both the sibling correlation and the exponential distribution of differences between sisters.

The two transition version of the Transition Probability model turned out to provide a remarkably good *quantitative* description of cell cycle variability in continuously cycling cells using just two parameters (the two transition probabilities), both of which were fixed in advance by the observed cell cycle statistics (standard deviation of cycle times, the mean or standard deviation of differences between sibling cycle times, and the sibling correlation), rather than curve-fitting (Brooks et al., 1980, 1983; Brooks, 1981, 1985). But, the nature of process L was never identified; early hopes that it might correspond to the biogenesis of mitotic centers (centrosomes) were not fulfilled (Brooks and Richmond, 1983; Alvey, 1985). In addition, many alternative models of cell cycle variability have been proposed and kinetics alone are insufficient to distinguish between them (Castor, 1980; Cooper, 1982; Yao, 2014; Arata and Takagi, 2019). Also lacking at the time was any plausible biological basis for the random transitions. Accordingly, transition probability models (and other kinetic models of the cell cycle) largely fell from view in favor of efforts to understand the molecular basis of cell cycle control. However, developments in systems biology have shown how interacting networks of continuous processes containing positive or double-negative feedback loops can generate bistability and ultrasensitivity, switching abruptly and irreversibly from one steady state to another with minimal perturbation (Thron, 1997; Aguda and Tang, 1999; Qu et al., 2003; Novák and Tyson, 2004; Yao et al., 2008; Stallaert et al., 2019). Such behavior could well provide an explanation for probabilistic transitions in terms of the now established molecular players in cell cycle control. A reconsideration of the two-transition model therefore seems timely.

Early studies indicated that the A state transition of the original Smith and Martin transition probability model – if it existed – must be located very close (within an hour or so) to the start of S phase (Brooks, 1977). A good case can be made that this transition corresponds to the abrupt and irreversible inactivation of APC/C^CDH1^. This event occurs at very different times in different cells (Cappell et al., 2016, 2018; Chung et al., 2019). It is triggered by the rise in CDK/E,A activity, the onset of which also shows a great deal of variability in timing between individual cells (Spencer et al., 2013; Overton et al., 2014; Arora et al., 2017; Barr et al., 2017; Moser et al., 2018). In cycling cells, the timing of the rise in CDK/E,A activity depends on the level of CDK4,6/D activity immediately after mitosis, which in turn depends on mitogenic signaling or replication stress in the mother cell (Spencer et al., 2013; Arora et al., 2017; Barr et al., 2017; Yang et al., 2017; Chung et al., 2019; Min et al., 2020). This means that sister cells are in a similar state at birth and likely to reach the point when CDK/E,A activity starts to rise at the same time, accounting for much (possibly all) of the correlation between sister cell cycle times. However, subsequent activation of the APC/C^CDH1^ switch is likely to occur at different times in sister cells due to stochastic variation. It seems possible that such differences in APC/C^CDH1^ inactivation account for the majority of variation between siblings in the timing of S phase entry, and subsequently mitosis. For continuously cycling cells, the differences between sister cell cycle times are an almost perfect exponential distribution (the so-called β-curve), providing the most compelling evidence for the Smith & Martin A state transition, as already discussed). It would therefore be of considerable interest to know whether differences in the timing of APC/C^CDH1^ inactivation in siblings are also exponentially distributed and identical to the differences in sister cell cycle times (the β-curve).

Although evidence for one random transition in the cell cycle remains good (β-curves), that for a second transition was always much less secure (Brooks, 1985). When the differences in sibling cycle times are subtracted, the left-over variation in cycle times within the population is well described by an exponential distribution (Brooks et al., 1980). However, it is likely that almost any other skewed distribution could fit just as well (Brooks, 1985). For cells re-entering the cycle from quiescence, it is clear that there is a great deal of population heterogeneity in the level of mitogenic stimulus required, with some cells much more sensitive than others. Transition probability models provide no insight into this heterogeneity. However, stochastic activation of a bistable RB-E2F switch, coupled with a variable switching threshold within the population (Kwon et al., 2017) due to known heterogeneity in the level of CDK-inhibitors such as p27 (Hitomi et al., 2006), is an attractive possibility.

Although the RB-E2F switch is widely accepted as the basis of the Restriction Point, as already discussed, in many types of continuously cycling cells, the majority are post-Restriction Point from birth, in so far as they continue on to S phase without further need of mitogenic stimulation (Spencer et al., 2013; Schwarz et al., 2018). The apparent lack of requirement for an RB-E2F switch can be explained if the level of CDK4,6/D activity is sufficiently high to stimulate enough active E2F (i.e., above the threshold required) to generate self-sustaining levels of cyclin E, from the start of G1. The level of CDK4,6/D activity after mitosis will depend on the extent of p21 and/or p27 expression, together with the amount of cyclin D protein inherited from the mother cell (Min et al., 2020). The levels of p21, p27 and cyclin D at the start of G1 clearly vary from cell to cell, but will be inherited more or less equally by sister cells, accounting for the sibling correlation. However, there is no obvious role for the RB-E2F bistable switch, or any other probabilistic transition in the mother cell cycle, in explaining the post-Restriction Point state of daughter cells from birth. Evidently, the RB-E2F switch is not an obligatory feature of each and every cell cycle but a special case applicable to cells re-entering the cycle from quiescence, or to cells born with a level of CDK4,6/D activity below the threshold required to achieve self-sustaining levels of cyclin E without mitogens (the CDK/E,A^*low*^ cohort).

In conclusion, evidence for one random transition in the cell cycle (the Smith and Martin “A” transition, located very close to the start of S phase, continues to be compelling, and it seems very plausible that it could correspond to the APC/C^CDH1^ inactivation switch. For continuously cycling cells, the existence of a second random transition (the “Q” transition of the modified model; Brooks et al., 1980), no longer seems likely, despite the excellent quantitative predictions of the model. For quiescent cells responding to mitogens, a good case can be made that the RB/E2F switch is the basis of the Restriction Point. Nevertheless, as discussed in an earlier section, stochastic noise in switching seems to be a less important contributor to variability than factors leading to differences in the threshold for switching between cells (Kwon et al., 2017), in accounting for the variation in growth factor sensitivity within a population.

## Other Sources of Variation in the Cell Cycle

If there is no probabilistic transition in the mother cell determining levels of p21, p27, and cyclin D at the start of G1 in daughter cells, then understanding the reasons why these vary between cells becomes important. It is already established that p21 levels reflect replication stress or unrepaired DNA damage in the mother cell, indicating a purely deterministic contribution to cell cycle variability (Arora et al., 2017; Barr et al., 2017; Yang et al., 2017). Factors regulating p27 expression are less well understood, other than that levels are generally low in rapidly cycling cells and rise when cells become quiescent (Coats et al., 1996; Hitomi et al., 2006). However, in contrast to non-transformed cells, rapidly cycling HeLa cells, which lack functioning RB-family proteins, have high levels of p27 in G1 (Barr et al., 2016), perhaps substituting for the lack of an intact RB-E2F network. Further studies of the factors generating variability in p27 expression at the level of single cells, and the contribution of such variability to cell cycle timing, would seem to be a priority.

As for variability in the amount of cyclin D inherited by daughter cells, this is determined by differences in translational capacity at the end of G2 in mother cells (Min et al., 2020). Translational capacity is a reflection of the number of ribosomes per cell, at least in part, and is therefore an indicator of cell size. The bigger the cell, the greater will be the total amount of cyclin D made in any moment in time, even though the translation rate per ribosome is likely to be similar between cells of different size. Since cyclin D is a nuclear protein, it will be concentrated in the nucleus. Bigger mother cells would therefore be expected to achieve a higher *nuclear* concentration of cyclin D than small cells, a difference that would be passed on to daughter cells. In turn, the higher nuclear levels of cyclin D in large daughters would accelerate their transit through G1, compared to small daughters. Cyclin D levels may therefore provide a link between translational capacity – and hence cell size – and cell cycle timing, potentially explaining (for mammalian cells) the widely observed inverse relationship between cell size and cell cycle time (Prescott, 1956; Fantes, 1977; Johnston et al., 1977; Miyata et al., 1978; Shields et al., 1978). A similar role in linking translation rate to the speed of transit through G1 has also been proposed for Cln3 (a functional homolog of cyclin D) in budding yeast (Polymenis and Schmidt, 1997; Litsios et al., 2019).

If the variation in cyclin D abundance at mitosis is due primarily to variation in cell size, then the faster transit of large daughters through G1 (due to high cyclin D), compared to small daughters, would narrow the dispersion of size by the time of the next mitosis. However, stochastic variation in the timing of the APC/C^CDH1^ inactivation switch, would generate renewed variation in cell size at division by affecting cycle length. These two conflicting processes – one decreasing size variance and the other increasing it – may help to explain how distributions of cell size remain stable over time under steady state conditions. Of course, many other factors are involved in the regulation of cell size but further consideration of this important topic is beyond the scope of this article. Instead, the reader is referred to Zatulovskiy and Skotheim (2020) for an excellent recent review.

## Concluding Remarks

As reviewed here, the recent technical innovations in live-cell imaging have revolutionized the study of cell cycle regulation and have brought renewed attention to the existence of cell cycle variability. Ostensibly identical cells clearly undergo critical transitions at different ages and with the focus on individual cells, this variability becomes difficult to ignore. The parallel developments in systems biology in understanding the role of positive-feedback loops in generating molecular switches have also provided invaluable insight into how cell cycle transitions might be controlled (Thron, 1997; Aguda and Tang, 1999; Qu et al., 2003; Novák and Tyson, 2004; Yao et al., 2008; Stallaert et al., 2019). One such bistable switch – APC/C^CDH1^ inactivation – has emerged as the most likely candidate for the point of no return in the mammalian cell cycle (Cappell et al., 2018), and noisy activation of the switch may well prove to be the basis of the elusive Smith and Martin random transition. Should this not turn out to be the case, then the origin of the exponential distribution of differences between sister cell cycle times would return as an important unanswered question.

For cell cycles as a whole, the variability is well-described by two random transitions (Brooks et al., 1980). However, no obvious candidate for the second transition has yet emerged. Instead, this additional variability, shared by sister cells, is likely to be a composite of several sources, of which cell size may be one of the most important. Cell size, as a determinant of translational capacity, influences the amount of cyclin D and CDK4,6 activity inherited by daughter cells, and the timing of the next cell cycle. In addition, nuclear size, again usually shared by sister cells, seems likely to determine the time taken to respond to the inducers of DNA synthesis present once the cell is committed to enter S phase.

As for the Restriction Point – the moment when cell cycle progress becomes independent of mitogenic stimulation – this can no longer be regarded as an obligatory and irreversible decision point in each and every cell cycle that all cells must pass through. Rather, for many cell types, the majority of rapidly cycling cells are already mitogen-independent from birth. Such cells inherit a level of CDK4,6/D activity above the threshold needed to generate sufficient cyclin E to maintain RB hyperphosphorylation in the absence of further mitogenic stimulation. However, cells born with a level of CDK4,6/D activity below the threshold lose RB hyperphosphorylation and return to a state of mitogen dependence for further progress though the cell cycle. For these, and for long-term quiescent cells, the Restriction Point concept remains valid. Indeed, stochastic activation of the RB-E2F switch may contribute to some of the variability in their subsequent re-entry into the cell cycle, though a bigger contribution seems to come from heterogeneity in the switching threshold, most likely due to differences between cells in the levels of CDK inhibitors (p27, p21). As for the notion that passage of the Restriction Point is strictly irreversible, this now requires qualification. Although it is not reversible with normal, physiological interventions (such as growth factor removal), pharmacological blocking of CDK4,6/D and (probably) CDK2/E activity (indirectly) leads to a loss of RB hyperphosphorylation and a return to mitogen dependence (Cappell et al., 2016). This is not possible after the APC/C^CDH1^ inactivation transition.

It is to be expected that further refinements of live-cell imaging techniques will lead to ever fuller understanding of why cell cycles are so variable. Nevertheless, it is worth emphasizing that this will require great precision in determining the timing of intracellular events. Classical cell biology approaches have led to the conclusion that most cell cycle variability is in G1 and that the duration of S + G2 is relatively constant (Sisken and Morasca, 1965; Tobey, 1973; Shields et al., 1978; Brooks et al., 1983; Zetterberg and Larsson, 1985). In contrast, recent live-cell imaging studies using a fluorescent PCNA reporter have concluded that G1, S, and G2 are all independently variable (Chao et al., 2019). However, this rests on noise-free determination of entry into and exit from S phase. These transitions are assessed by changes in the granularity of PCNA fluorescence (nuclear foci). In most cases, this is unambiguous, but with a few cells it is difficult to say precisely when S phase begins and ends (especially the former). Indeed, this is apparent in Figure 2E of Chao et al. (2017) where a few cells in S phase have very few PCNA foci. Such measurement uncertainty would increase the apparent variance in phase length estimates, possibly explaining the disparity with the older work. Measurement noise may also be an issue in determining CDK/E,A activity. This involves determining the ratio of nuclear to cytoplasmic levels of the DHB reporter. As such, the estimate is affected by the variance in both the nuclear and cytoplasmic measurements, which widens the confidence intervals. Added to this, the measurements may be affected by cell motility. As cells move, they round up and flatten out periodically, to varying degrees. This is likely to affect the thickness of cytoplasm in the region of the ring around the nucleus used for quantitating reporter fluorescence. This could lead to variation in the fluorescence signal independent of any change in CDK/E,A activity. Such noise could well account for some of the differences in activity traces shown by different cells. Quantitating, or better, eliminating any such measurement noise is likely to become important in the discrimination between models.

It has often been said that cell division is too important to be left to chance yet variability seems to be embedded in the very fabric of cell cycle control. There are very good reasons why it should be. In free-living, single-cell organisms, cell cycle entry is controlled by nutrient availability. When nutrients are restored to starved cells, it would be undesirable for all of them to commit immediately to cell cycle re-entry, in case the restoration of nutrients is short-lived. If starvation conditions rapidly return, this could compromise survival of cells already committed to cell division. It is clearly advantageous for the timing of commitment to vary between cells, particularly in response to suboptimal conditions, where a graded response is needed in the population such that some cells respond and others do not. In the case of multicellular organisms, cell division is controlled less by nutrient supply than by growth factor signaling in the context of tissue homeostasis. After wounding, new cells are required to repair the deficit, but it is important that not all cells in the tissue respond to the stimulus, to avoid an overshoot. This is not just a matter of having a localized stimulus in the proximity of the wound. In partial hepatectomy, cell division resumes throughout the liver remnant, not just at the cut edge, yet the response is proportional to need (the extent of hepatectomy). Ensuring a graded response to the level of mitogens is clearly of fundamental importance. *A priori*, this requires either an inherently probabilistic response to mitogenic stimuli, with probability proportional to the stimulus, or the generation of actual differences between cells in the threshold for response – or most likely both, as has been discussed here. Uncovering all the mechanisms involved clearly remains an important goal. In recent years, progress in understanding the molecular details of critical cell cycle transitions within individual cells has been impressive. There is now every reason to hope that in the very near future, a full, molecular understanding of the origins of the variability will be forthcoming.

## Author Contributions

RB wrote and completed the review.

## Conflict of Interest

The author declares that the research was conducted in the absence of any commercial or financial relationships that could be construed as a potential conflict of interest.

## Publisher’s Note

All claims expressed in this article are solely those of the authors and do not necessarily represent those of their affiliated organizations, or those of the publisher, the editors and the reviewers. Any product that may be evaluated in this article, or claim that may be made by its manufacturer, is not guaranteed or endorsed by the publisher.

## References

[B1] Aguda B. D. Tang Y. (1999). The kinetic origins of the restriction point in the mammalian cell cycle. *Cell Prolif.* 32 321–335. 10.1046/j.1365-2184.1999.3250321.x 10619492 PMC6726334

[B2] Alvey P. L. (1985). An investigation of the centriole cycle using 3T3 and CHO cells. *J. Cell Sci.* 78 147–162. 10.1242/jcs.78.1.147 4093469

[B3] Anders L. Ke N. Hydbring P. Choi Y. Widlund H. Chick J. (2011). A Systematic Screen for CDK4/6 Substrates Links FOXM1 Phosphorylation to Senescence Suppression in Cancer Cells. *Cancer Cell* 20 620–634. 10.1016/j.ccr.2011.10.001 22094256 PMC3237683

[B4] Arata Y. Takagi H. (2019). Quantitative Studies for Cell-Division Cycle Control. *Frontiers in Physiology* 10:1022. 10.3389/fphys.2019.01022 PMC6713215 31496950

[B5] Arora M. Moser J. Phadke H. Basha A. A. Spencer S. L. (2017). Endogenous Replication Stress in Mother Cells Leads to Quiescence of Daughter Cells. *Cell Reports* 19 1351–1364. 10.1016/j.celrep.2017.04.055 28514656 PMC5533606

[B6] Bachman K. E. Blair B. G. Brenner K. Bardelli A. Arena S. Zhou S. (2004). 21 (WAF1/CIP1) Mediates the Growth Response to TGF-b in Human Epithelial Cells. *null* 3 221–225. 10.4161/cbt.3.2.666 14726675

[B7] Barr A. R. Cooper S. Heldt F. S. Butera F. Stoy H. Mansfeld J. (2017). DNA damage during S-phase mediates the proliferation-quiescence decision in the subsequent G1 via 21 expression. *Nature Communications* 8 14728. 10.1038/ncomms14728 PMC5364389 28317845

[B8] Barr A. R. Heldt F. S. Zhang T. Bakal C. Novák B. (2016). A Dynamical Framework for the All-or-None G1/S Transition. *Cell Systems* 2 27–37. 10.1016/j.cels.2016.01.001 27136687 PMC4802413

[B9] Bashir T. Dorrello N. V. Amador V. Amador V. F. Guardavaccaro D. F. Pagano M. (2004). Control of the SCF(Skp2-Cks1) ubiquitin ligase by the APC/C(Cdh1) ubiquitin ligase. *Nature* 428 190–193. 10.1038/nature02330 15014502

[B10] Bertoli C. Skotheim J. M. de Bruin R. A. M. (2013). Control of cell cycle transcription during G1 and S phases. *Nature Reviews Molecular Cell Biology* 14 518–528. 10.1038/nrm3629 23877564 PMC4569015

[B11] Blow J. J. Dutta A. (2005). Preventing re-replication of chromosomal DNA. *Nature Reviews Molecular Cell Biology* 6 476–486. 10.1038/nrm1663 15928711 PMC2688777

[B12] Bouchard C. Thieke K. Maier A. Saffrich R. Hanley-Hyde J. Ansorge W. (1999). Direct induction of cyclin D2 by Myc contributes to cell cycle progression and sequestration of 27. *EMBO J.* 18 5321–5333. 10.1093/emboj/18.19.5321 10508165 PMC1171602

[B13] Bracken A. P. Ciro M. Cocito A. Helin K. (2004). E2F target genes: unraveling the biology. *Trends in Biochemical. Sciences.* 29 409–417. 10.1016/j.tibs.2004.06.006 15362224

[B14] Brooks R. F. (1975). The kinetics of serum-induced initiation of DNA synthesis in BHK21/c13 cells, and the influence of exogenous adenosine. *J. Cell. Physiol.* 86 369–377. 10.1002/jcp.1040860409 1194373

[B15] Brooks R. F. (1976). Regulation of the fibroblast cell cycle by serum. *Nature* 260 248–250. 10.1038/260248a0 942592

[B16] Brooks R. F. (1977). Continuous protein synthesis is required to maintain the probability of entry into S phase. *Cell* 12 311–317. 10.1016/0092-8674(77)90209-4 902318

[B17] Brooks R. F. (1981). “Variability in the cell cycle and the control of proliferation,” in *The cell cycle*, ed. John P. C. L. (Cambridge: Cambridge University Press), 35–61.

[B18] Brooks R. F. (1985). “The transition probability model: successes, limitations and deficiences,” in *Temporal Order*, eds Rensing L. Jaeger N. I. (Berlin: Springer-Verlag), 304–314. 10.1007/978-3-642-70332-4_49

[B19] Brooks R. F. Richmond F. N. (1983). Microtubule-organizing centres during the cell cycle of 3T3 cells. *J. Cell Sci.* 61 231–245. 10.1242/jcs.61.1.231 6350330

[B20] Brooks R. F. Riddle P. N. (1988a). Differences in growth factor sensitivity between individual 3T3 cells arise at high frequency: possible relevance to cell senescence. *Expl. Cell Res.* 174 378–387. 10.1016/0014-4827(88)90308-4 3338495

[B21] Brooks R. F. Riddle P. N. (1988b). The 3T3 cell cycle at low proliferation rates. *J. Cell Sci.* 90 601–612. 10.1242/jcs.90.4.601 3253297

[B22] Brooks R. F. Bennett D. C. Smith J. A. (1980). Mammalian cell cycles need two random transitions. *Cell* 19 493–504. 10.1016/0092-8674(80)90524-3 7357616

[B23] Brooks R. F. Richmond F. N. Riddle P. N. Richmond K. M. V. (1984). Apparent heterogeneity in the response of quiescent Swiss 3T3 cells to serum growth factors: implications for the transition probability model and parallels with “cellular senescence” and “competence”. *J. Cell. Physiol.* 121 341–350. 10.1002/jcp.1041210211 6333428

[B24] Brooks R. F. Riddle P. N. Richmond F. N. Marsden J. (1983). The G1 distribution of “G1-less” V79 Chinese hamster cells. *Expl. Cell Res.* 148 127–142. 10.1016/0014-4827(83)90193-3 6628553

[B25] Burk R. R. (1970). One-step growth cycle for BHK21-13 hamster fibroblasts. *Exp. Cell Res* 63 309–316. 10.1016/0014-4827(70)90218-1 5530916

[B26] Cappell S. D. Chung M. Jaimovich A. Spencer S. L. Meyer T. (2016). Irreversible APCCdh1 Inactivation Underlies the Point of No Return for Cell-Cycle Entry. *Cell* 166 167–180. 10.1016/j.cell.2016.05.077 27368103 PMC6649667

[B27] Cappell S. D. Mark K. G. Garbett D. Pack L. R. Rape M. Meyer T. (2018). EMI1 switches from being a substrate to an inhibitor of APC/CCDH1 to start the cell cycle. *Nature* 558 313–317. 10.1038/s41586-018-0199-7 29875408 PMC6035873

[B28] Castor L. N. (1980). A G1 rate model accounts for the cell-cycle kinetics attributed to “transition probability”. *Nature* 287 857–859. 10.1038/287857a0 6159544

[B29] Chao H. X. Fakhreddin R. I. Shimerov H. K. Kedziora K. M. Kumar R. J. Perez J. (2019). Evidence that the human cell cycle is a series of uncoupled, memoryless phases. *Mol. Syst. Biol.* 15 e8604. 10.15252/msb.20188604 PMC6423720 30886052

[B30] Chao H. X. Poovey C. E. Privette A. A. Grant G. D. Chao H. Y. Cook J. G. (2017). Orchestration of DNA Damage Checkpoint Dynamics across the Human Cell Cycle. *Cell Systems* 5 445–459. 10.1016/j.cels.2017.09.015 29102360 PMC5700845

[B31] Cheng M. Olivier P. Diehl J. A. Fero M. Roussel M. F. Roberts J. M. (1999). The p21Cip1 and p27Kip1 CDK “inhibitors” are essential activators of cyclin D-dependent kinases in murine fibroblasts. *EMBO J.* 18 1571–1583. 10.1093/emboj/18.6.1571 10075928 PMC1171245

[B32] Choi Y. J. Anders L. (2014). Signaling through cyclin D-dependent kinases. *Oncogene* 33 1890–1903. 10.1038/onc.2013.137 23644662

[B33] Choudhury R. Bonacci T. Arceci A. Lahiri D. Mills C. A. Kernan J. L. (2016). APC/C and SCFcyclin F Constitute a Reciprocal Feedback Circuit Controlling S-Phase Entry. *Cell Reports* 16 3359–3372. 10.1016/j.celrep.2016.08.058 27653696 PMC5111906

[B34] Chu I. Sun J. Arnaout A. Kahn H. Hanna W. Narod S. (2007). 27 Phosphorylation by Src Regulates Inhibition of Cyclin E-Cdk2. *Cell* 128 281–294. 10.1016/j.cell.2006.11.049 17254967 PMC1961623

[B35] Chung M. Liu C. Yang H. W. Köberlin M. S. Cappell S. D. Meyer T. (2019). Transient Hysteresis in CDK4/6 Activity Underlies Passage of the Restriction Point in G1. *Molecular Cell* 76 1–12. 31543423 10.1016/j.molcel.2019.08.020 PMC7189330

[B36] Coats S. Flanagan M. Nourse J. Roberts J. (1996). Requirement of p27Kip1 for restriction point control of the fibroblast cell cycle. *Science* 272 877–880. 10.1126/science.272.5263.877 8629023

[B37] Cobrinik D. (2005). Pocket proteins and cell cycle control. *Oncogene* 24 2796–2809. 10.1038/sj.onc.1208619 15838516

[B38] Cooper S. (1982). The continuum model: statistical implications. *J Theor. Biol* 94 783–800. 10.1016/0022-5193(82)90078-9 7078225

[B39] Coudreuse D. Nurse P. (2010). Driving the cell cycle with a minimal CDK control network. *Nature* 468 1074–1079. 10.1038/nature09543 21179163

[B40] Fantes P. A. (1977). Control of cell size and cycle time in Schizosaccharomyces pombe. *J Cell Sci* 24 51–67. 10.1242/jcs.24.1.51 893551

[B41] Fragkos M. Ganier O. Coulombe P. Mechali M. (2015). DNA replication origin activation in space and time. *Nat Rev. Mol. Cell Biol* 16 360–374. 10.1038/nrm4002 25999062

[B42] Fujimaki K. Yao G. (2020). Cell dormancy plasticity: quiescence deepens into senescence through a dimmer switch. *Physiological Genomics* 52 558–562. 10.1152/physiolgenomics.00068.2020 32986540

[B43] Fujimaki K. Li R. Chen H. Della Croce K. Zhang H. H. Xing J. (2019). Graded regulation of cellular quiescence depth between proliferation and senescence by a lysosomal dimmer switch. *PNAS* 116 22624. 10.1073/pnas.1915905116 31636214 PMC6842626

[B44] Ginzberg M. B. Chang N. D’Souza H. Patel N. Kafri R. Kirschner M. W. (2018). Cell size sensing in animal cells coordinates anabolic growth rates and cell cycle progression to maintain cell size uniformity. *Elife* 7 e26957. 10.7554/eLife.26957 PMC6031432 29889021

[B45] Guiley K. Z. Stevenson J. W. Lou K. Barkovich K. J. Kumarasamy V. Wijeratne T. U. (2019). 27 allosterically activates cyclin-dependent kinase 4 and antagonizes palbociclib inhibition. *Science* 366 eaaw2106. 10.1126/science.aaw2106 31831640 PMC7592119

[B46] Harrigan J. A. Belotserkovskaya R. Coates J. Dimitrova D. S. Polo S. E. Bradshaw C. R. (2011). Replication stress induces 53BP1-containing OPT domains in G1 cells. *J Cell Biol* 193 97–108. 10.1083/jcb.201011083 21444690 PMC3082192

[B47] Heldt F. S. Barr A. R. Cooper S. Bakal C. Novák B. (2018). A comprehensive model for the proliferation-quiescence decision in response to endogenous DNA damage in human cells. *PNAS* 115 2532. 10.1073/pnas.1715345115 29463760 PMC5877942

[B48] Hitomi M. Yang K. Guo Y. Fretthold J. Harwalkar J. Stacey D. (2006). p27Kip1 and Cyclin Dependent Kinase 2 Regulate Passage Through the Restriction Point. *Cell Cycle* 5 2281–2289. 10.4161/cc.5.19.3318 16969133

[B49] Hola M. Castleden S. Howard M. Brooks R. F. (1994). Initiation of DNA synthesis by nuclei from scrape-ruptured quiescent mammalian cells in high speed supernatants of Xenopus egg extracts. *J. Cell Sci.* 107 3045–3053. 10.1242/jcs.107.11.3045 7699004

[B50] Hola M. Howard M. Nawaz F. N. Castleden S. Brooks R. F. (1996). Individual nuclei differ in their sensitivity to the cytoplasmic inducers of DNA synthesis: implications for the origin of cell cycle variability. *Expl. Cell Res.* 229 350–359. 10.1006/excr.1996.0380 8986618

[B51] Holley R. W. Kiernan J. A. (1968). “Contact inhibition” of cell division in 3T3 cells. *Proc. Natl. Acad. Sci U. S. A* 60 300–304. 10.1073/pnas.60.1.300 5241531 PMC539117

[B52] Hsu J. Y. Reimann J. D. R. Sørensen C. S. Lukas J. Jackson P. K. (2002). E2F-dependent accumulation of hEmi1 regulates S phase entry by inhibiting APCCdh1. *Nature Cell Biology* 4 358–366. 10.1038/ncb785 11988738

[B53] Johnson A. Skotheim J. M. (2013). Start and the restriction point. *Current. Opinion. in Cell Biology* 25 717–723. 10.1016/j.ceb.2013.07.010 23916770 PMC3836907

[B54] Johnson D. G. Schwarz J. K. Cress W. D. Nevins J. R. (1993). Expression of transcription factor E2F1 induces quiescent cells to enter S phase. *Nature* 365 349–352. 10.1038/365349a0 8377827

[B55] Johnston G. C. Pringle J. R. Hartwell L. H. (1977). Coordination of growth with cell division in the yeast Sacharomyces cerevisiae. *Expl. Cell Res.* 105 79–98. 10.1016/0014-4827(77)90154-9 320023

[B56] Klein E. A. Assoian R. K. (2008). Transcriptional regulation of the cyclin D1 gene at a glance. *J. Cell Sci.* 121 3853. 10.1242/jcs.039131 19020303 PMC4545630

[B57] Koundrioukoff S. Carignon S. Técher H. Letessier A. Brison O. Debatisse M. (2013). Stepwise Activation of the ATR Signaling Pathway upon Increasing Replication Stress Impacts Fragile Site Integrity. *PLoS Genetics* 9:e1003643. 10.1371/journal.pgen.1003643 23874235 PMC3715430

[B58] Kwon J. S. Everetts N. J. Wang X. Wang W. Croce K. D. Xing J. (2017). Controlling Depth of Cellular Quiescence by an Rb-E2F Network Switch. *Cell Reports* 20 3223–3235. 10.1016/j.celrep.2017.09.007 28954237 PMC6571029

[B59] Lee T. J. Yao G. Bennett D. C. Nevins J. R. You L. (2010). Stochastic E2F Activation and Reconciliation of Phenomenological Cell-Cycle Models. *PLoS Biol* 8:e1000488. 10.1371/journal.pbio.1000488 20877711 PMC2943438

[B60] Leone G. Sears R. Huang E. Rempel R. Nuckolls F. Park C. H. (2001). Myc Requires Distinct E2F Activities to Induce S Phase and Apoptosis. *Molecular Cell* 8 105–113. 10.1016/s1097-2765(01)00275-1 11511364

[B61] Leung J. Y. Ehmann G. L. Giangrande P. H. Nevins J. R. (2008). A role for Myc in facilitating transcription activation by E2F1. *Oncogene* 27 4172–4179. 10.1038/onc.2008.55 18345030

[B62] Litsios A. Huberts D. H. E. W. Terpstra H. M. Guerra P. Schmidt A. Buczak K. (2019). Differential scaling between G1 protein production and cell size dynamics promotes commitment to the cell division cycle in budding yeast. *Nature Cell Biology* 21 1382–1392. 10.1038/s41556-019-0413-3 31685990

[B63] Lu Z. Hunter T. (2010). Ubiquitylation and proteasomal degradation of the 21(Cip1), p27(Kip1) and p57(Kip2) CDK inhibitors. *Cell Cycle* 9 2342–2352. 10.4161/cc.9.12.11988 20519948 PMC3319752

[B64] Lukas C. Savic V. Bekker-Jensen S. Doil C. Neumann B. Sølvhøj Pedersen R. (2011). 53BP1 nuclear bodies form around DNA lesions generated by mitotic transmission of chromosomes under replication stress. *Nature Cell Biology* 13 243–253. 10.1038/ncb2201 21317883

[B65] Mailand N. Diffley J. F. X. (2005). CDKs Promote DNA Replication Origin Licensing in Human Cells by Protecting Cdc6 from APC/C-Dependent Proteolysis. *Cell* 122 915–926. 10.1016/j.cell.2005.08.013 16153703

[B66] Malumbres M. Barbacid M. (2009). Cell cycle, CDKs and cancer: a changing paradigm. *Nat Rev Cancer* 9 153–166. 10.1038/nrc2602 19238148

[B67] Min M. Rong Y. Tian C. Spencer S. (2020). Temporal integration of mitogen history in mother cells controls proliferation of daughter cells. *Science* 368 1261–1265. 10.1126/science.aay824 32241885 PMC8363187

[B68] Minor P. D. Smith J. A. (1974). Explanation of degree of correlation of sibling generation times in animal cells. *Nature.* 248 241–243. 10.1038/248241a0 4206605

[B69] Mittnacht S. (1998). Control of pRB phosphorylation. *Current Opinion. in Genetics & Development* 8 21–27. 10.1016/s0959-437x(98)80057-9 9529601

[B70] Miyata H. Miyata M. Ito M. (1978). The cell cycle in the fission yeast, Schizosaccharomyces pombe. I. Relationship between cell size and cycle time. *Cell Struct. & Funct.* 3 39–46. 10.1247/csf.3.39

[B71] Moreno A. Carrington J. T. Albergante L. Al Mamun M. Haagensen E. J. Komseli E. S. (2016). Unreplicated DNA remaining from unperturbed S phases passes through mitosis for resolution in daughter cells. *PNAS* 113 E5757–E5764. 27516545 10.1073/pnas.1603252113 PMC5047195

[B72] Moser J. Miller I. Carter D. Spencer S. L. (2018). Control of the Restriction Point by Rb and 21. *PNAS* 115 E8219. 10.1073/pnas.1722446115 PMC6126733 30111539

[B73] Narasimha A. M. Kaulich M. Shapiro G. S. Choi Y. J. Sicinski P. Dowdy S. F. (2014). Cyclin D activates the Rb tumor suppressor by mono-phosphorylation. *Elife* 3 e02872. 10.7554/eLife.02872 PMC4076869 24876129

[B74] Nassrally M. S. Lau A. Wise K. John N. Kotecha S. Lee K. L. (2019). Cell cycle arrest in replicative senescence is not an immediate consequence of telomere dysfunction. *Mechanisms of Ageing and Development* 179 11–22. 10.1016/j.mad.2019.01.009 30710559

[B75] Newport J. Y. (1996). Organization of DNA into foci during replication. *Curr. Opin. Cell Biol.* 8 365–368. 10.1016/s0955-0674(96)80011-1 8743888

[B76] Novák B. Tyson J. J. (2004). A model for restriction point control of the mammalian cell cycle. *Journal of Theoretical. Biology* 230 563–579. 10.1016/j.jtbi.2004.04.039 15363676

[B77] Overton K. W. Spencer S. L. Noderer W. L. Meyer T. Wang C. L. (2014). Basal 21 controls population heterogeneity in cycling and quiescent cell cycle states. *Proc. Natl. Acad. Sci. USA* 111 E4386–E4393. 25267623 10.1073/pnas.1409797111 PMC4205626

[B78] Pardee A. B. (1974). A restriction point for control of normal animal cell proliferation. *Proc. Natl. Acad. Sci. U. S. A.* 71 1286–1290. 10.1073/pnas.71.4.1286 4524638 PMC388211

[B79] Pennycook B. R. Barr A. R. (2020). Restriction point regulation at the crossroads between quiescence and cell proliferation. *FEBS Lett.* 594 2046–2060. 10.1002/1873-3468.13867 32564372

[B80] Peters J. M. (2006). The anaphase promoting complex/cyclosome: a machine designed to destroy. *Nature Reviews Molecular Cell Biology* 7 644–656. 10.1038/nrm1988 16896351

[B81] Planas-Silva M. D. Weinberg R. A. (1997). The restriction point and control of cell proliferation. *Current. Opinion. in Cell Biology.* 9 768–772. 10.1016/s0955-0674(97)80076-2 9425340

[B82] Polymenis M. Schmidt E. V. (1997). Coupling of cell division to cell growth by translational control of the G1 cyclin CLN3 inΓÇëyeast. *Genes & Development* 11 2522–2531. 10.1101/gad.11.19.2522 9334317 PMC316559

[B83] Prescott D. M. (1956). Relation between cell growth and cell divison. II. The effect of cell size on cell growth rate and generation time in Amoeba proteus. *Expl. Cell Res.* 11 86–98. 10.1016/0014-4827(56)90192-6 13356829

[B84] Qu Z. MacLellan W. R. Weiss J. N. (2003). Dynamics of the Cell Cycle: Checkpoints, Sizers, and Timers. *Biophysical Journal* 85 3600–3611. 10.1016/s0006-3495(03)74778-x 14645053 PMC1303665

[B85] Rodier G. Coulombe P. Tanguay P. L. Boutonnet C. Meloche S. (2008). Phosphorylation of Skp2 regulated by CDK2 and Cdc14B protects it from degradation by APC(Cdh1) in G1 phase. *EMBO J.* 27 679–691. 10.1038/emboj.2008.6 18239684 PMC2262036

[B86] Sadasivam S. DeCaprio J. A. (2013). The DREAM complex: master coordinator of cell cycle-dependent gene expression. *Nat Rev. Cancer* 13 585–595. 10.1038/nrc3556 23842645 PMC3986830

[B87] Sage J. Mulligan G. J. Attardi L. D. Miller A. Chen S. Williams B. (2000). Targeted disruption of the three Rb-related genes leads to loss of G(1) control and immortalization. *Genes Dev.* 14 3037–3050. 10.1101/gad.843200 11114892 PMC317090

[B88] Sakaue-Sawano A. Kurokawa H. Morimura T. Hanyu A. Hama H. Osawa H. (2008). Visualizing Spatiotemporal Dynamics of Multicellular Cell-Cycle Progression. *Cell* 132 487–498. 10.1016/j.cell.2007.12.033 18267078

[B89] Schade A. E. Oser M. G. Nicholson H. E. DeCaprio J. A. (2019). Cyclin D−CDK4 relieves cooperative repression of proliferation and cell cycle gene expression by DREAM and RB. *Oncogene* 38 4962–4976. 10.1038/s41388-019-0767-9 30833638 PMC6586519

[B90] Schwarz C. Johnson A. Kõivomägi M. Zatulovskiy E. Kravitz C. J. Doncic A. (2018). A Precise Cdk Activity Threshold Determines Passage through the Restriction Point. *Molecular Cell* 69 253–264. 10.1016/j.molcel.2017.12.017 29351845 PMC5790185

[B91] Segeren H. A. van Rijnberk L. M. Moreno E. Riemers F. M. van Liere E. A. Yuan R. (2020). Excessive E2F Transcription in Single Cancer Cells Precludes Transient Cell-Cycle Exit after DNA Damage. *Cell Reports* 33 108449. 10.1016/j.celrep.2020.108449 33264622

[B92] Sherr C. J. (1995). D-type cyclins. *Trends in biochemical. sciences* 20 187–190. 7610482 10.1016/s0968-0004(00)89005-2

[B93] Sherr C. J. (2019). Surprising regulation of cell cycle entry. *Science* 366 1315. 10.1126/science.aaz4043 31831659

[B94] Shields R. (1977). Transition probability and the origin of variation in the cell cycle. *Nature* 267 704–707. 10.1038/267704a0 876390

[B95] Shields R. (1978). Further evidence for a random transition in the cell cycle. *Nature.* 273 755–758. 10.1038/273755a0 661983

[B96] Shields R. Smith J. A. (1977). Cells regulate their proliferation through alterations in transition probability. *J. Cell. Physiol.* 91 345–356. 10.1002/jcp.1040910304 558988

[B97] Shields R. Brooks R. F. Riddle P. N. Capellaro D. F. Delia D. (1978). Cell size, cell cycle and transition probability in mouse fibroblasts. *Cell* 15 469–474. 10.1016/0092-8674(78)90016-8 569024

[B98] Sicinska E. Aifantis I. Le Cam L. Swat W. Borowski C. Yu Q. (2003). Requirement for cyclin D3 in lymphocyte development and T cell leukemias. *Cancer Cell* 4 451–461. 10.1016/s1535-6108(03)00301-5 14706337

[B99] Sigl R. Wandke C. Rauch V. Kirk J. Hunt T. Geley S. (2009). Loss of the mammalian APC/C activator FZR1 shortens G1 and lengthens S phase but has little effect on exit from mitosis. *J. Cell Sci.* 122 4208. 10.1242/jcs.054197 19861496

[B100] Sisken J. E. Morasca J. (1965). Intrapopulation kinetics of the mitotic cycle. *J. Cell Biol.* 25 179–189. 10.1083/jcb.25.2.179 19866661 PMC2106641

[B101] Smith J. A. Martin L. (1973). Do cells cycle? *Proc. Natl. Acad. Sci. U. S. A.* 70 1263–1267. 4515625 10.1073/pnas.70.4.1263 PMC433472

[B102] Spencer S. L. Cappell S. D. Tsai F. C. Overton K. W. Wang C. L. Meyer T. (2013). The Proliferation-Quiescence Decision Is Controlled by a Bifurcation in CDK2 Activity at Mitotic Exit. *Cell* 155 369–383. 10.1016/j.cell.2013.08.062 24075009 PMC4001917

[B103] Stallaert W. Kedziora K. M. Chao H. X. Purvis J. E. (2019). Bistable switches as integrators and actuators during cell cycle progression. *FEBS Lett* 593 2805–2816. 10.1002/1873-3468.13628 31566708 PMC7881439

[B104] Sun W. H. Hola M. Baldwin N. Pedley K. Brooks R. F. (2001). Heterogeneity in nuclear transport does not affect the timing of DNA synthesis in quiescent mammalian cells induced to replicate in Xenopus egg extracts. *Cell Prolif.* 34 55–67. 10.1046/j.1365-2184.2001.00196.x 11284919 PMC6495702

[B105] Sun W. H. Hola M. Pedley K. Tada S. Blow J. J. Todorov I. T. (2000). The replication capacity of intact mammalian nuclei in Xenopus egg extracts declines with quiescence, but the residual DNA synthesis is independent of Xenopus MCM proteins. *J. Cell Sci.* 113 683–695. 10.1242/jcs.113.4.683 10652261

[B106] Takahashi Y. Rayman J. B. Dynlacht B. D. (2000). Analysis of promoter binding by the E2F and pRB families in vivo: distinct E2F proteins mediate activation and repression. *Genes & Development* 14 804–816. 10766737 PMC316494

[B107] Temin H. M. (1971). Stimulation by serum of multiplication of stationary chicken cells. *J Cell Physiol* 78 161–170. 10.1002/jcp.1040780202 5167847

[B108] Thron C. D. (1997). Bistable biochemical switching and the control of the events of the cell cycle. *Oncogene* 15 317–325. 10.1038/sj.onc.1201190 9233766

[B109] Tobey R. A. (1973). “Production and Characterization of Mammalian Cells Reversibly Arrested in G1 by Growth in Isoleucine-Deficient Medium,” in *Methods in Cell Biology*, ed. Prescott D. M. (Cambridge, MA: Academic Press), 67–112. 10.1016/s0091-679x(08)60048-5 4585084

[B110] Todaro G. J. Lazar G. K. Green H. (1965). The initiation of cell division in a contact-inhibited mammalian cell line. *J Cell Physiol* 66 325–333. 10.1002/jcp.1030660310 5884360

[B111] Topacio B. R. Zatulovskiy E. Cristea S. Xie S. Tambo C. S. Rubin S. M. (2019). Cyclin D-Cdk4,6 Drives Cell-Cycle Progression via the Retinoblastoma Protein’s C-Terminal Helix. *Molecular Cell* 74 758–770. 10.1016/j.molcel.2019.03.020 30982746 PMC6800134

[B112] Wei W. Ayad N. G. Wan Y. Zhang G. J. Kirschner M. W. Kaelin W. G. (2004). Degradation of the SCF component Skp2 in cell-cycle phase G1 by the anaphase-promoting complex. *Nature* 428 194–198. 10.1038/nature02381 15014503

[B113] Weinberg R. A. (2013). *The Biology of Cancer. Garland.*

[B114] Wiebusch L. Hagemeier C. (2010). 53- and p21-dependent premature APC/C−Cdh1 activation in G2 is part of the long-term response to genotoxic stress. *Oncogene* 29 3477–3489. 10.1038/onc.2010.99 20383190

[B115] Wu L. Timmers C. Maiti B. Saavedra H. I. Sang L. Chong G. T. (2001). The E2F1−3 transcription factors are essential for cellular proliferation. *Nature* 414 457–462. 10.1038/35106593 11719808

[B116] Yang H. W. Chung M. Kudo T. Meyer T. (2017). Competing memories of mitogen and 53 signalling control cell-cycle entry. *Nature* 549 404–408. 10.1038/nature23880 28869970 PMC6544019

[B117] Yao G. (2014). Modelling mammalian cellular quiescence. *Interface Focus* 4 20130074. 10.1098/rsfs.2013.0074 24904737 PMC3996586

[B118] Yao G. Lee T. J. Mori S. Nevins J. R. You L. (2008). A bistable Rb-E2F switch underlies the restriction point. *Nature Cell Biology* 10 476–482. 10.1038/ncb1711 18364697

[B119] Yen A. Pardee A. B. (1979). Role of nuclear size in cell growth initiation. *Science* 204 1315. 10.1126/science.451539 451539

[B120] Yuan X. Srividhya J. De Luca T. Lee J. H. Pomerening J. R. (2014). Uncovering the role of APC-Cdh1 in generating the dynamics of S-phase onset. *MBoC* 25 441–456. 10.1091/mbc.e13-08-0480 24356446 PMC3923637

[B121] Zatulovskiy E. Skotheim J. M. (2020). On the Molecular Mechanisms Regulating Animal Cell Size Homeostasis. *Trends in Genetics* 36 360–372. 10.1016/j.tig.2020.01.011 32294416 PMC7162994

[B122] Zetterberg A. Larsson O. (1985). Kinetic analysis of regulatory events in G1 leading to proliferation or quiescence of Swiss 3T3 cells. *Proc. Natl. Acad. Sci. U. S. A.* 82 5365–5369. 10.1073/pnas.82.16.5365 3860868 PMC390569

